# Dynamics and genetic diversification of *Escherichia coli* during experimental adaptation to an anaerobic environment

**DOI:** 10.7717/peerj.3244

**Published:** 2017-05-03

**Authors:** Thomas J. Finn, Sonal Shewaramani, Sinead C. Leahy, Peter H. Janssen, Christina D. Moon

**Affiliations:** 1 Grasslands Research Centre, AgResearch Ltd, Palmerston North, New Zealand; 2 New Zealand Institute for Advanced Study, Massey University, Auckland, New Zealand; 3 Department of Biochemistry, University of Otago, Dunedin, New Zealand; 4 Department of Molecular and Cell Biology, University of Connecticut, Storrs, CT, United States of America

**Keywords:** Anaerobic, *Escherichia coli*, Adaptation, Fermentation, Genomics, Experimental evolution

## Abstract

**Background:**

Many bacteria are facultative anaerobes, and can proliferate in both anoxic and oxic environments. Under anaerobic conditions, fermentation is the primary means of energy generation in contrast to respiration. Furthermore, the rates and spectra of spontaneous mutations that arise during anaerobic growth differ to those under aerobic growth. A long-term selection experiment was undertaken to investigate the genetic changes that underpin how the facultative anaerobe, *Escherichia coli*, adapts to anaerobic environments.

**Methods:**

Twenty-one populations of *E. coli* REL4536, an aerobically evolved 10,000th generation descendent of the *E. coli* B strain, REL606, were established from a clonal ancestral culture. These were serially sub-cultured for 2,000 generations in a defined minimal glucose medium in strict aerobic and strict anaerobic environments, as well as in a treatment that fluctuated between the two environments. The competitive fitness of the evolving lineages was assessed at approximately 0, 1,000 and 2,000 generations, in both the environment of selection and the alternative environment. Whole genome re-sequencing was performed on random colonies from all lineages after 2,000-generations. Mutations were identified relative to the ancestral genome, and based on the extent of parallelism, traits that were likely to have contributed towards adaptation were inferred.

**Results:**

There were increases in fitness relative to the ancestor among anaerobically evolved lineages when tested in the anaerobic environment, but no increases were found in the aerobic environment. For lineages that had evolved under the fluctuating regime, relative fitness increased significantly in the anaerobic environment, but did not increase in the aerobic environment. The aerobically-evolved lineages did not increase in fitness when tested in either the aerobic or anaerobic environments. The strictly anaerobic lineages adapted more rapidly to the anaerobic environment than did the fluctuating lineages. Two main strategies appeared to predominate during adaptation to the anaerobic environment: modification of energy generation pathways, and inactivation of non-essential functions. Fermentation pathways appeared to alter through selection for mutations in genes such as *nadR, adhE, dcuS/R*, and *pflB*. Mutations were frequently identified in genes for presumably dispensable functions such as toxin-antitoxin systems, prophages, virulence and amino acid transport. Adaptation of the fluctuating lineages to the anaerobic environments involved mutations affecting traits similar to those observed in the anaerobically evolved lineages.

**Discussion:**

There appeared to be strong selective pressure for activities that conferred cell yield advantages during anaerobic growth, which include restoring activities that had previously been inactivated under long-term continuous aerobic evolution of the ancestor.

## Introduction

Advances in our understanding of adaptation to novel environments, and the population dynamics and genetic changes that underpin these, have been significantly enhanced by long-term experimental evolution (LTEE) studies (Barrick et al., 2009; Blount, Borland & Lenski, 2008; Lenski et al., 1991). Such studies have typically employed highly defined and reproducible experimental conditions with regard to growth media, temperature and pH. Only a few have examined the impact of oxygen availability on adaptation, where low oxygen contrasts have been used (Manché, Notley-McRobb & Ferenci, 1999; Puentes-Téllez et al., 2013). Facultatively anaerobic organisms, such as *E. coli*, grow in both aerobic and anaerobic environments. They are metabolically versatile, in particular, possessing distinct energy generating systems required to thrive in each environment (Smith & Neidhardt, 1983). In the presence of oxygen, energy is conserved in the form of ATP via aerobic respiration which involves glycolysis, the tricarboxylic acid (TCA) cycle, and the electron transport chain (Gunsalus, 1992; Ingledew & Poole, 1984) with oxygen as the terminal electron acceptor. However, the natural environment of *E. coli* is the lower gut, which is predominantly anaerobic, though microaerobic conditions are also apparent and where oxygen availability may fluctuate (He et al., 1999; Jones et al., 2007). Under anaerobic conditions, fermentation is the principle process for generating ATP in the absence of oxygen, or other terminal electron acceptors. Fermentation employs glycolysis in conjunction with substrate-level phosphorylation from 1,3-bisphosphoglycerate, phosphoenolpyruvate, and acetyl-phosphate (Clark, 1989). The energy yields during growth in aerobic and anaerobic environments differ, with 26 ATP molecules generated per glucose during aerobic respiration (Kaleta et al., 2013; Haddock & Schairer, 1973). In contrast, anaerobic fermentation of glucose by *E. coli* is less productive, yielding a maximum of three molecules of ATP per glucose, depending on which fermentation pathways are used (Gunsalus & Park, 1994). In some facultative anaerobes, organic compounds such as citrate can be metabolised under anaerobic conditions and thus increase energy yields when they are co-metabolised with other substrates e.g., glucose (Bott, 1997; Lütgens & Gottschalk, 1980).

While higher energy yields are obtained during aerobic respiration, natural by-products from this process include reactive oxygen species (ROS), which are mutagenic and can result in distinct mutational spectra (Maki, 2002; Sakai et al., 2006; Shewaramani et al., 2017). Despite this, the spontaneous mutation rate of anaerobically grown *E. coli* is higher than that of aerobically grown *E. coli* (Sakai et al., 2006; Shewaramani et al., 2017), although the mutagenic agents in the anaerobic environment are not fully understood. It is hypothesised that the different mutation spectra and rates imposed by each environment are likely to impact the evolutionary trajectory of organisms during adaptation.

Independent *E. coli* populations that are allowed to evolve in a controlled experimental environment have been shown to take different adaptive paths to achieve increased fitness (Elena & Lenski, 2003; Lenski & Travisano, 1994). Functions accounting for these adaptations include DNA supercoiling (Crozat et al., 2005), modification of the stringent response (Cooper, Rozen & Lenski, 2003), increased substrate specificity (Cooper et al., 2001) and substrate switching (Blount, Borland & Lenski, 2008). The rate at which a population adapts to an environment, and the path(s) taken, are also influenced by the degree to which the population is exposed to an environment. Prolonged exposure to a constant environment is likely to promote the evolution of specialists that have high fitness in the environment of selection (Cooper & Lenski, 2000), but decreased fitness in alternative environments. This may arise due to selection for beneficial mutations in the environment that have detrimental effects in alternative environments (Elena & Lenski, 2003). However, varying exposure to a range of environments will likely result in the evolution of generalists (Kassen, 2002; Kassen & Bell, 1998), which may evolve unique adaptations that are beneficial in the range of environments to which it is exposed.

In the present study, we sought to better understand the adaptation of *E. coli* to environments that vary in oxygen availability, to provide insights into evolution in its natural habitat, which consists of passage through gastrointestinal tracts. Experimental growth of *E. coli* was modelled on the prominent LTEE study of Richard Lenski (Lenski et al., 1991), using an aerobically evolved derivative of his ancestral strain, and maintained on the same minimal glucose medium. However, parallel populations were also evolved under strictly anaerobic growth conditions, and under a treatment that fluctuated exposure to both aerobic and anaerobic environments. We report the genes and mutations that are important for adaptation to anaerobic environments, and provide new insights into the evolutionary pathways involved in adaptation during aerobic and anaerobic growth.

## Methods

### Strains and growth media

The ancestral strain used in this study was *E. coli* strain REL4536 (Barrick et al., 2009), an aerobically evolved 10,000th generation descendent of the *E. coli* B strain, REL606 (Lenski et al., 1991). Cultures were propagated aerobically in a biosafety cabinet, and anaerobically in an anaerobic chamber (Coy Laboratories, Grass Lake, MI, USA) with a 95% CO_2_:5% H_2_ atmosphere. DM25 media (Lenski et al., 1991) based on Davis minimal media (DM; Carlton & Brown, 1981) were used to propagate populations in the experimental evolution experiment. For anaerobic preparation of DM25, the base DM broth (Carlton & Brown, 1981) was boiled and cooled to room temperature while bubbling CO_2_ through it. Resazurin (0.00002% wt/vol) was added as an oxygen indicator, and the medium was sterilised by autoclaving. Before use, l-cysteine HCl (0.025% wt/vol), was added as a reducing agent. Anaerobically-prepared magnesium sulfate (0.01% wt/vol, final concentration), thiamine (0.002% wt/vol final concentration), and glucose (0.0025% wt/vol final concentration) were filter sterilised, then added. DM1000 media used for the generation of a neutrally-marked strain for competition assays was prepared like DM25 but contained 0.1% (wt/vol) glucose.

DM agar plates were prepared as for DM media, with the addition of bacteriological grade 1.6% (wt/vol) agar and 0.4% (wt/vol) glucose, combined after sterilisation via autoclaving. DM plates were used for the isolation of randomly-selected clones for genome sequencing, where aerobic plates were incubated in air, and anaerobic plates were incubated in AnaeroJar™ gas canisters (Oxoid, Cheshire, UK), with oxygen indicator strips (Oxoid) to monitor oxygen conditions. The plates were placed in the jars in the anaerobic chamber, so the gas composition was the same as in the anaerobic chamber.

Lysogeny broth (LB) and LB agar plates (Bertani, 1951) were used for cultivating clones for genomic DNA extraction, and enumerating bacteria after competitive fitness experiments, respectively.

### Long-term evolution experiment

A single randomly-selected colony of *E. coli* REL4536 was inoculated into 9.90 mL DM25, and incubated with shaking overnight at 37 °C to generate an ancestral REL4536 culture. From this, 21 independent *E. coli* populations were established for the LTEE, with seven populations propagated under each treatment regime: aerobic growth (AE), anaerobic growth (AN) and growth with temporally fluctuating exposure to aerobic and anaerobic environments (FL). Establishment of the long-term AE and FL populations took place aerobically, with inoculation of 10 µL of the ancestral culture into 990 µL of aerobic DM25 in 24-well plates (Becton, Dickinson and Co., Sparks, MD, USA). An uninoculated (medium-only) control well was included alongside the seven populations in each treatment to monitor for cross-contamination. The AN cultures were established in anaerobic DM25 medium, and cultures grown anaerobically in anaerobic gas boxes (AnaeroPack^®^ 2.5 L, Mitsubishi Gas Company Inc., Tokyo Japan). All cultures were incubated at 37 °C with orbital shaking at 150 rpm, and propagated by daily serial subculture of 1/100th volume of the previous day’s culture into fresh growth medium. The FL treatment was alternated daily between aerobic and anaerobic growth. The lineages were propagated for approximately 2,000 generations (ca. 300 days, at ∼6.67 generations per day). To monitor for external contaminants, growth within the medium-only wells was monitored, and routine testing with coliphage T5 and T6 was performed (Lenski et al., 1991). Aliquots of evolved lineages were stored in 70% (vol/vol) glycerol at −85 °C every two weeks.

### Competitive fitness assays and calculating fitness

The fitness of the evolved populations relative to the ancestral strain was assessed both aerobically and anaerobically after approximately 1,000 and 2,000 generations. Spontaneous arabinose-utilising (Ara^+^) strains were generated from the ancestral strain to competitively assess fitness, following the approach used by Lenski et al. (1991). However, these displayed poor fitness in the anaerobic environment relative to the unmarked strain. Therefore, a rifampicin (Rif) resistant spontaneous mutant of REL4536 was obtained by plating cells on DM agar plates containing 100 µg/mL rifampicin (DM+Rif). The fitness of the Rif^r^ competitor relative to REL4536 was 0.97 ± 0.05 and 1.04 ± 0.05 under aerobic and anaerobic growth conditions, respectively, and deemed selectively neutral and used for all competitive fitness assays performed in this study.

For competition assays, competing strains were acclimated to the growth conditions by inoculating frozen stocks into wells of 24-well plates containing 1-ml volumes of either DM25 for competing strains, or DM25+Rif for the Rif^r^ competitor. Cultures were grown overnight at 37 °C with 150 rpm orbital shaking. At 0 h, 10 µL of each competitor culture was mixed at an estimated cell ratio of 1:1 (evolved strain:Rif^r^ ancestral strain) in fresh DM25 media, resulting in a 1 ml total competition volume. Competitors were enumerated at 0 and 24 h by plating serial dilutions in duplicate onto both LB and LB+Rif plates, followed by overnight incubation at 37 °C. The ancestor (Rif^r^) cell densities were calculated directly from growth on LB+Rif plates, while the cell density of the competitor was derived from subtracting the Rif^r^ cell density from the total cell density (on LB plates). The Malthusian parameters (*m*) for each competitor was calculated as: $m=ln \left( \frac{\text{Cell density at 24}\mathrm{h}}{\text{Cell density at 0}\mathrm{h}} \right) $. Relative fitness (*ω*) of the evolved strain relative to the ancestral was calculated as $\omega = \frac{\mathrm{m} \left( \mathrm{Evolved} \right) }{\mathrm{m} \left( \mathrm{Ancestral} \right) } $ (Lenski et al., 1991). Anaerobic competitive fitness assays were carried out for anaerobic and fluctuating lineages as above, except using anaerobic practices as described previously.

### Whole genome sequencing and mutation detection

To identify mutations that arose during the LTEE, single, randomly selected clones were isolated from each of the 21 lineages at 2,000 generations. Genomic DNA was extracted from clones cultured in LB using a Qiagen Genomic-tip 100/G kit (Qiagen, Hilden, Germany). DNA was sequenced on an Illumina HiSeq 2000 instrument using paired-end sequencing with 90 bp reads of 500 bp paired-end libraries (BGI, Shenzhen, China). Approximately 1 Gb of clean sequence data (filtered to remove reads containing ≥10% unreadable bases, ≥20% low quality (≤Q20) bases, adapter contamination or duplicate read-pairs) were obtained for each sample, representing over 200 × fold coverage over each genome. The FastQC tool (Andrews, 2010), was used to assess FASTQ file quality.

The reference *E. coli* REL4536 sequence was obtained by manually editing the *E. coli* REL606 genome sequence (GenBank accession number NC_012967.1) to incorporate mutations that arose in REL4536 relative to REL606 (Barrick et al., 2009). Mutations in the genomes of the evolved strains were identified relative to REL4536 using breseq v.014 (Barrick et al., 2009). Biological processes affected by mutations in evolved lineages were inferred using the PANTHER protein (and gene) evolutionary and functional classification system with the “Functional classification viewed in gene list” analysis option (Mi et al., 2016). Genome sequence data generated in this study are available in NCBI BioProject PRJNA357967.

### Statistical analyses

Fitness data for competition assays held in both aerobic and anaerobic environments were checked for normal distribution using a Shapiro–Wilk test (Shapiro & Wilk, 1965). To determine if lineages had adapted to the aerobic or anaerobic environments, repeated measurements analysis of variance (RM ANOVA) tests were performed, with relative fitness of evolved lineages in either environment as a response, and lineage and generation as factors. Fisher’s Least Significant Difference (LSD) post hoc tests were performed to identify which lineages had increased in relative fitness. Differences in the rates at which adaptation occurred were examined using paired *t*-tests. To identify if the different lineage treatments had statistically significant mutation types, single factor ANOVAs were performed followed by LSD post hoc tests to identify which lineages had significant differences. Comparisons of the proportion of mutations was analysed with a Fisher’s exact test, which is suitable for small sample sizes. Statistical analyses were performed in either GenStat (17th edition), Minitab (17th edition) or Microsoft Office Excel (version 14).

## Results and Discussion

### Growth dynamics of REL4536 in the anaerobic environment

**Figure 1:**
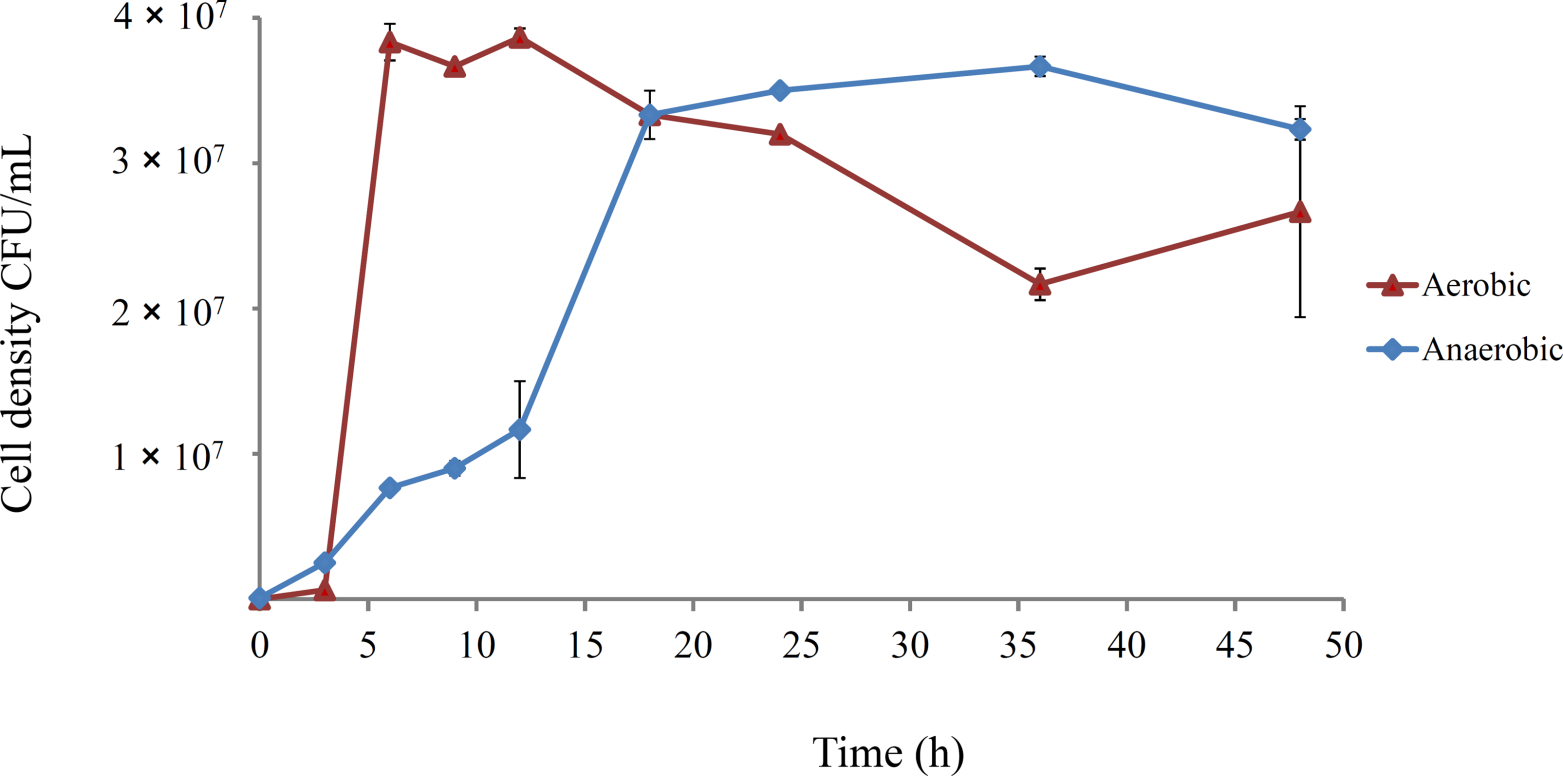
Growth course of *E. coli* REL4536 under aerobic and anaerobic conditions in DM25 media at 37°C. Data points represent mean values from three biological replicates. Error bars represent one standard error of the mean in each direction.

The growth dynamics of *E. coli* B strains in DM25 under aerobic conditions are well-documented (Lenski et al., 1998; Lenski et al., 1991; Vasi, Travisano & Lenski, 1994). However, anaerobic growth has not been well characterised, and was investigated for REL4536 in DM25 (Fig. 1).

Aerobically-grown cultures reached a maximum density of approximately 4.0 × 10^7^ CFU/mL within 6 h. In the anaerobic environment, the cell density reached approximately 3.5 × 10^7^ CFU/mL over 20 h, and had a lower growth rate than the aerobically grown cells, due to metabolic differences under each condition (Gray, Wimpenny & Mossman, 1966; Gunsalus & Park, 1994). However, the similar cell densities attained under the contrasting environments was unexpected, and likely due to the ability of *E. coli* to metabolise citrate when grown anaerobically (Blount, Borland & Lenski, 2008; Lütgens & Gottschalk, 1980; Vaughn et al., 1950) in DM25, which contains 1.7 mM citrate. REL4536 grew to a similar cell density in DM25 devoid of glucose (DM0), where citrate was the only carbon source available. Because both the aerobic and anaerobically grown cultures reached similar cell densities within 24 h (Fig. 1), serial sub-culturing was performed every 24 h in the LTEE of this study. Under this regime, the anaerobic lineages spent less time in stationary phase than the aerobic lineages as it was not practical to subculture more frequently (e.g., every 6–8 h). As such, interpretation of the data based on the different oxic treatments may be confounded by the differing lengths of time at stationary phase associated with each treatment. The aerobic lineages, being subject to longer periods of starvation, may have had more opportunity to generate growth advantage in stationary phase (GASP) mutations (Zambrano & Kolter, 1996). While such possibilities exist, GASP mutations are more commonly observed in cells that have been exposed to prolonged starvation over many days (Finkel, 2006). Furthermore, as significant fitness increases for the aerobic lineages were not observed (see below), the impact of the difference in stationary phase exposure between treatments was considered minimal.

### Fitness dynamics of evolved populations in aerobic and anaerobic environments

**Figure 2:**
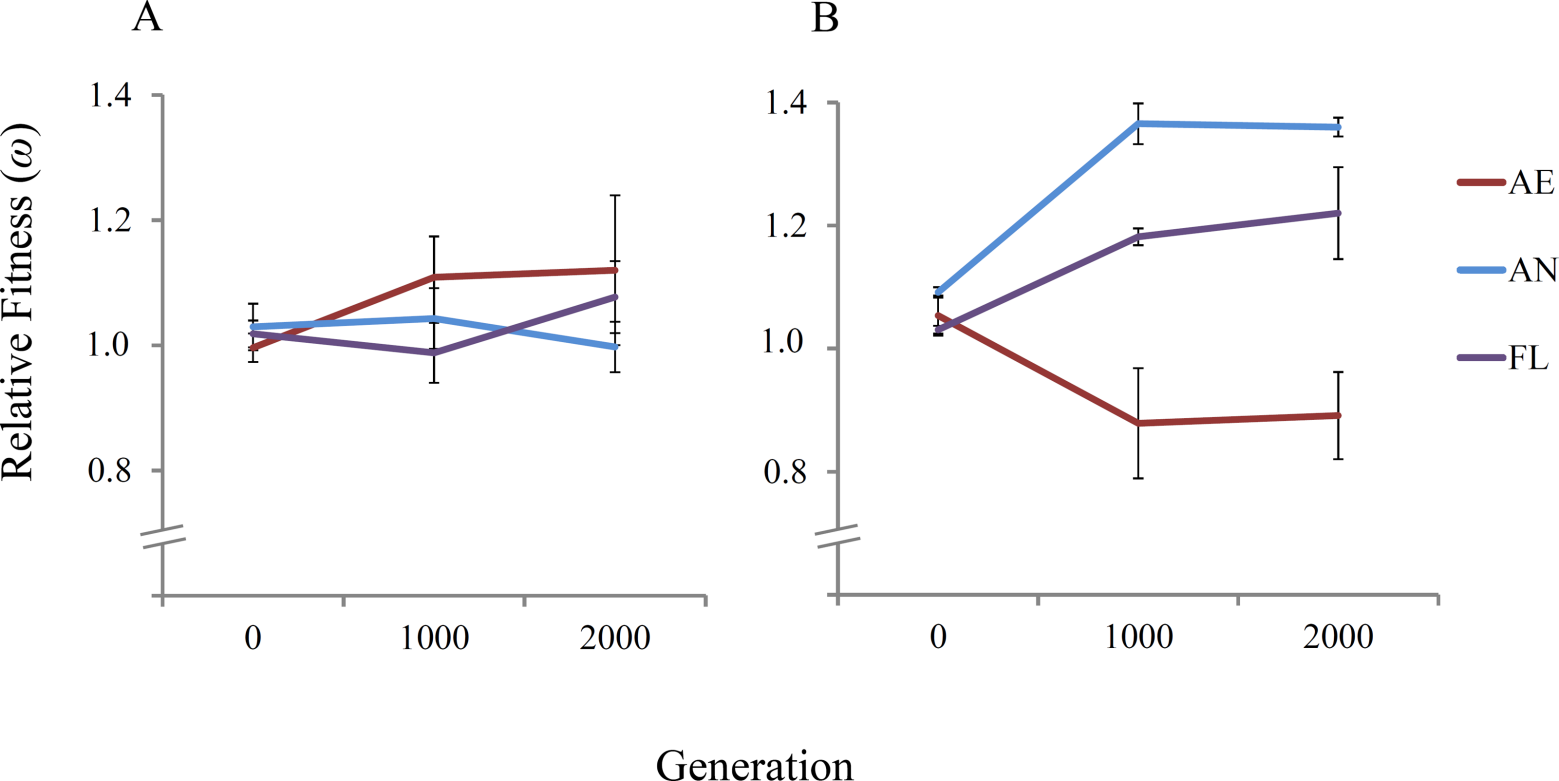
Mean relative fitness of lineages over 2,000 generations. Displayed are the mean relative fitness values of three randomly-selected lineages per treatment (AE2, AE3 and AE7; AN1, AN4 and AN6; and FL2, FL3 and FL7) relative to the Rif^r^ ancestor in (A) the aerobic environment and (B) the anaerobic environment. Error bars represent one standard error of the mean in each direction.

The 21 independent *E. coli* REL4536 lineages were evolved for approximately 2,000 generations over a period of 300 days under the AE, AN and FL treatments of this study. To assess the fitness dynamics of the evolved lineages, the fitness of three randomly-selected lineages per treatment were determined relative to a Rif^r^ ancestral strain at approximately 0, 1,000 and 2,000 generations in both aerobic and anaerobic environments (Fig. 2).

In the aerobic environment, significant increases in mean relative fitness were not observed among the tested lineages in any treatments over the 2,000 generations of evolution (RM ANOVA, *F*_(4,12)_ = 2.22, *P* = 0.163) (Table 1). The lack of fitness increase among the AE and FL lineages is expected because REL4536 had previously been adapted to the same medium under aerobic growth conditions for over 10,000 generations (Lenski et al., 1991; Barrick et al., 2009). No increase in mean fitness was reported among lineages during a similar 2,000 generation timeframe, where mean fitness was ∼1.77 at 10,000 generations and ∼1.72 at 12,000 generations (Barrick et al., 2009). When the AN lineages were competed in the aerobic environment, no significant change in fitness was seen, suggesting the ancestral fitness level in the aerobic environment was largely retained among anaerobic lineages. This may have arisen due to the substitution of mutations with pleiotropic effects during anaerobic adaptation, suggesting that beneficial mutations that occurred in the anaerobic environment were presumably neutral, weakly beneficial or weakly deleterious in the aerobic environment, and therefore, did not adversely affect AN lineage performance in the aerobic environment.

**Table 1:** RM ANOVA of fitness of lineages over 2,000 generations in aerobic and anaerobic conditions.

Source	Degrees of freedom	Aerobic		Anaerobic	
		*F*	*P*	*F*	*P*^a^
Lineage	(2, 12)	0.31	0.747	28.58	<0.001*
Generation	(2, 12)	1.47	0.273	4.13	0.045*
Generation × Lineage	(4, 12)	2.22	0.163	8.32	0.002*

**Notes.**

a Asterisk indicates statistical significance at *P* < 0.05.

In the anaerobic environment, there was a significant difference between the mean relative fitness of lineages over 2,000 generations (RM ANOVA, *F*_(4,12)_ = 8.32, *P* = 0.002) (Table 1). Fitness differences were detected at 1,000 generations, where AN and FL lineages had significantly higher mean fitness at 1,000 generations than at generation 0 (Table 2). The increased fitness of the AN and FL lineages under anaerobic conditions is consistent with rapid fitness increases observed by populations exposed to novel environments and suggests that many beneficial mutations were acquired in the first 1,000 generations of the experiment. Mean fitness increased from 1.091 to 1.408, ∼30% over the first 1,000 generations for the AN lineages (Table 2), which is in line with fitness increases reported in LTEE studies that expose populations to a novel environment (Puentes-Téllez et al., 2013; Lenski et al., 1991; Melnyk & Kassen, 2011). Furthermore, the rapid adaptation under anaerobic conditions may be facilitated by the elevated spontaneous mutation rate during anaerobic growth where it is almost double that for aerobic growth for REL4536 (Shewaramani et al., 2017) where higher mutation rates can lead to more rapid rates of fitness increase (Gerrish & Lenski, 1998).

**Table 2:** Fisher’s LSD test of mean fitness of lineages over 1,000 generations relative to initial mean fitness levels in the anaerobic environment.

Lineages	Mean fitness at generation 0 (*ω* ± SEM)	Mean fitness at generation 1,000 (*ω* ± SEM)	Difference^a^
AE	1.054 ± 0.032	0.879 ± 0.089	0.175*
AN	1.091 ± 0.008	1.408 ± 0.043	0.317*
FL	1.031 ± 0.006	1.182 ± 0.013	0.151*

**Notes.**

a Differences are significant if greater than 0.150, and are indicated with an asterisk.

The FL lineages displayed increased fitness in both environments (8% in the aerobic environment and 22% in the anaerobic environment), but only the fitness increases in the anaerobic environment were statistically significant (*P* < 0.05). Furthermore, fitness increases of the FL lineages in the anaerobic environment were not as great as for the AN specialists. This is consistent with evolutionary theory, and with other studies that have exposed experimental populations to novel environments in a variable manner (Kassen, 2002; Elena & Lenski, 2003; Ketola et al., 2013; Leroi, Lenski & Bennet, 1994). In the present study, FL lineage fitness increased by ∼18% in the first 1,000 generations, as compared to 11% in a similar set up where lineages were exposed to oxygen-rich and oxygen-poor conditions in a rich medium (Puentes-Téllez et al., 2013), and 20% where lineages were exposed to alternative sugar sources in a non-constant manner (Melnyk & Kassen, 2011).

### Mutation analysis of evolved lineages

To identify the full set of mutations that occurred during experimental evolution, whole genome sequencing of single randomly-selected 2,000 generation clones from each of the 21 lineages was performed. Relative to the REL4536 genome, 209 mutations were identified among the 21 genomes. Of these, 31, 94 and 84 were observed in the AE, AN and FL treatments, respectively (Tables S1, S2 and S3). Of the seven AN lineages, three of the randomly-selected sequenced clones (AN-4, AN-6 and AN-7) displayed a small colony morphology when incubated aerobically on solid LB medium. However, no mutations were found in common between these three genomes and so the genetic basis for this phenotype is unclear.

On average, AE, AN and FL lineages accumulated 4.4, 13.4 and 12 mutations per lineage respectively. When compared to other studies that exposed populations to novel environments, approximately six mutations per genome were identified within 2,000 generations during aerobic adaptation to minimal glucose medium (Barrick et al., 2009), and ∼11 mutations per genome observed within 2,000 generations during adaptation to an environment with elevated temperature (Tenaillon et al., 2012). Within the present study, presuming none of these mutations directly impacted mutation rate, the much greater number of mutations in the AN and FL lineage genomes compared to the AE likely reflects selection for mutations that are adaptive in the novel environment, as compared to the aerobic environment, which the ancestor had been extensively pre-adapted (Lenski et al., 1991). Furthermore, the spontaneous mutation rate during anaerobic growth is almost double that during aerobic growth for REL4536 (Shewaramani et al., 2017), thus providing greater genetic variation in anaerobically grown cells upon which selection can act to facilitate adaptation.

Overall, mutations were considered on three levels: (i) their effect on gene/protein function, (ii) their effect on biological processes and (iii) occurrence (parallelism) between lineages, implying an adaptive role.

**Figure 3:**
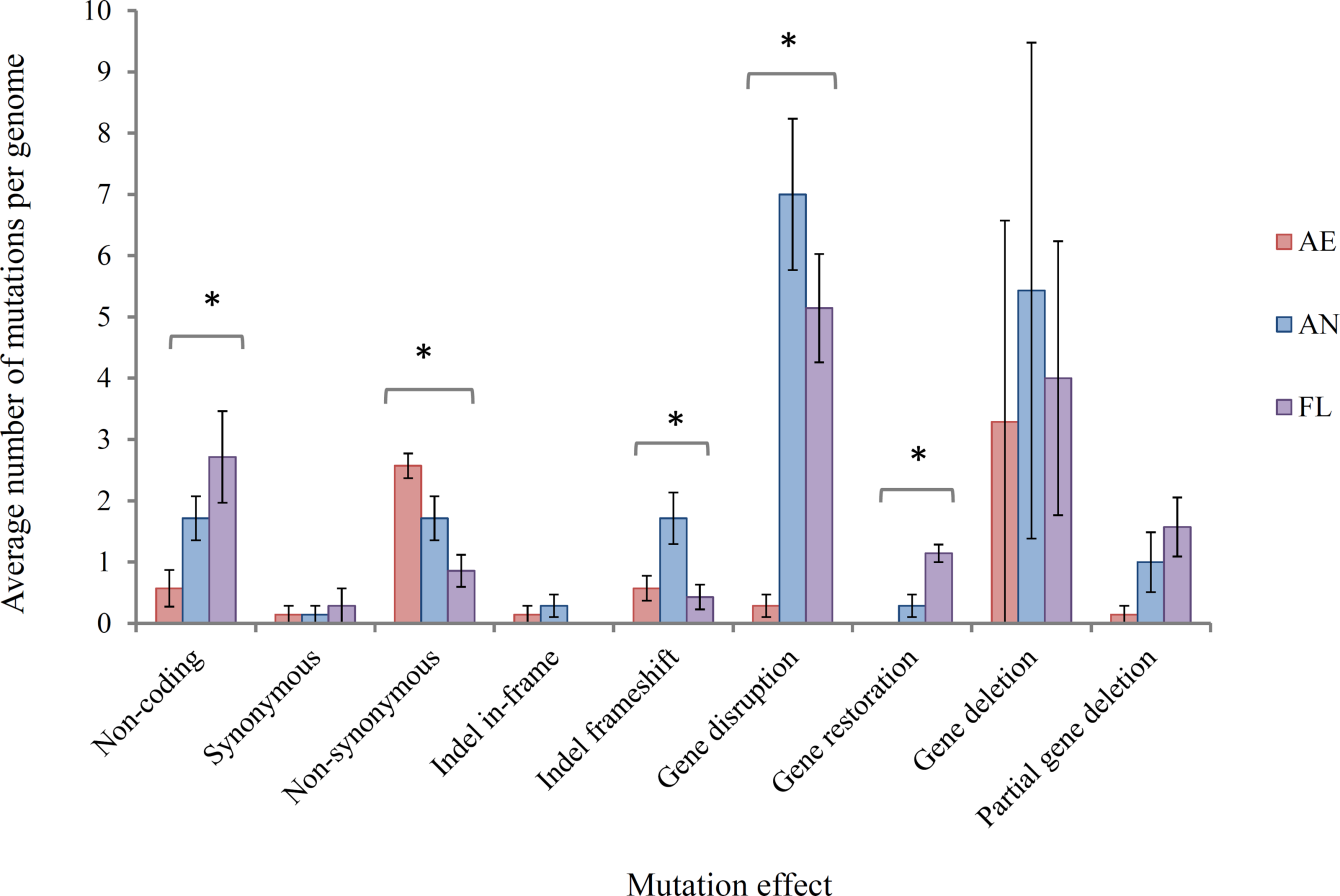
Average number of mutations per genome in lineages propagated under each treatment for 2,000 generations. Error bars represent one standard error of the mean of seven clones.^∗^ Significant at the *P* = 0.05 level, single factor ANOVA.

**(i) Effects of mutations at gene/protein level** At the level of the gene and protein function, mutations were classified, with predicted impacts on generating adaptive mutations, into the following main types: (a) non-coding DNA, i.e., mutations occurring in intergenic regions that may or may not impact the expression of adjacent genes, (b) synonymous SNPs, which are generally assumed not to alter gene function, (c) non-synonymous SNPs, which may impact gene function to varying degrees of severity from similar amino acid changes to gene truncation, (d) indel in-frame, which may result in altered gene function but does not result in frameshift, (e) indel frameshift, which likely disrupts gene function but, in cases, can restore the function of pseudogenes that have arisen through frameshift mutations, (f) insertion sequence (IS) element-mediated gene disruption, (g) gene restoration where the loss of an IS element restores a gene that had been previously disrupted, (h) complete gene deletion and (i) partial gene deletion. The average number of mutations per genome in each of these classes is shown in Fig. 3.

In this study, it was hypothesised that among the SNPs arising in AE evolved clones, an increase in G:C → T:A and A:T → C:G transversions would be expected due to ROS-mediated damage on DNA for aerobically grown clones relative to AN clones. However, these specific transversions were not found at a higher frequency (Fisher’s exact test, *P* > 0.05 in all cases), and could likely be explained by highly efficient repair mechanisms for these mutations in the aerobic environment (Shewaramani et al., 2017; Sakai et al., 2006).

**Figure 4:**
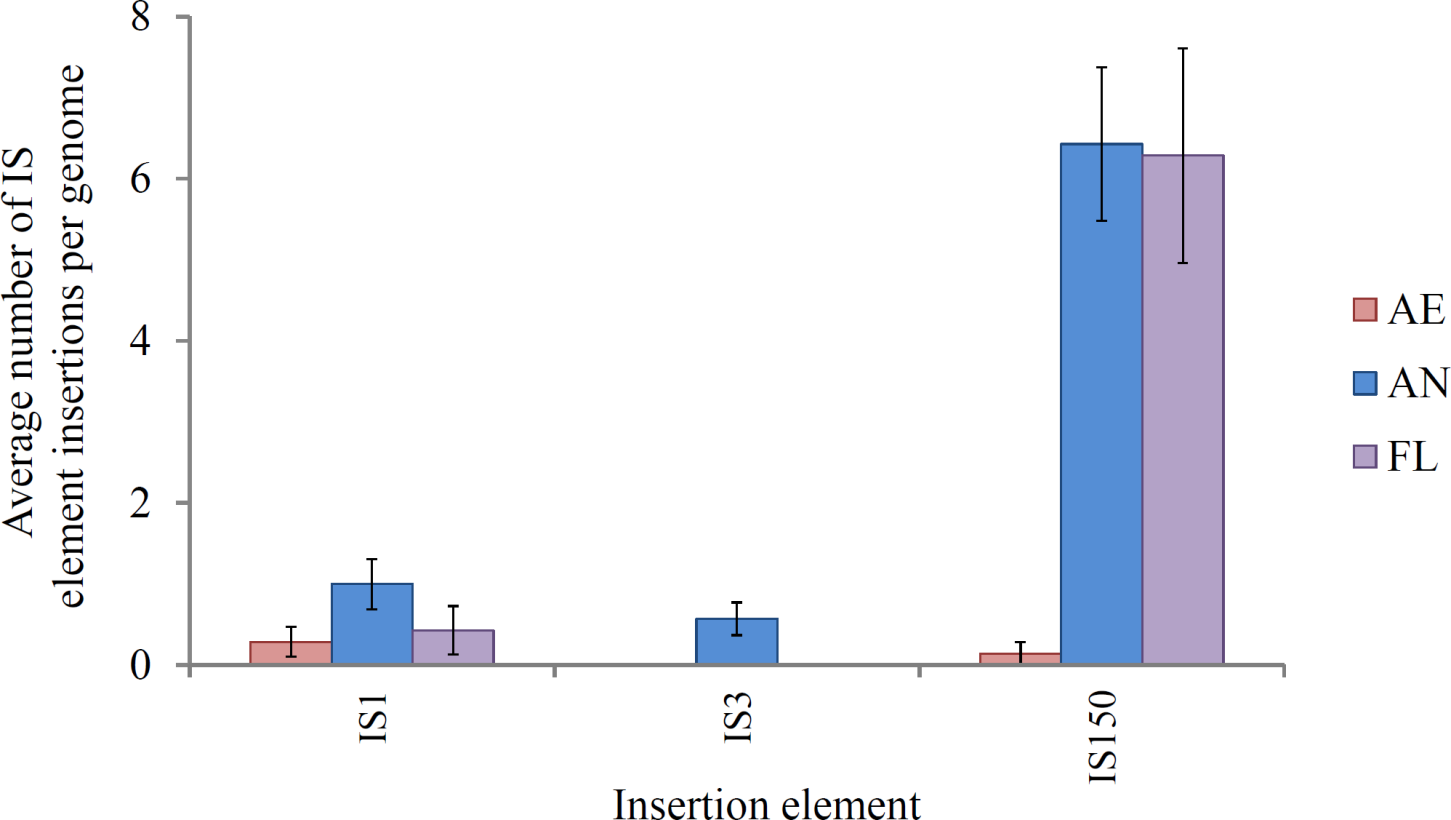
Average number of IS1, IS3 and IS150 insertions per genome in lineages propagated under each treatment for 2,000 generations. Error bars represent one standard error of the mean of seven clones.

IS-mediated mutations (gene disruptions, restorations and deletions) comprised the largest class of mutations in lineages exposed to the anaerobic environment, consistent with their high spontaneous mutation rate (approximately 8 × 10^−4^ mutations per genome/generation; Shewaramani et al., 2017). This suggests that this class of mutation is likely to play an important role in adaptation. IS element activity is a key feature of the *E. coli* B genome (Jeong et al., 2009; Papadopoulos et al., 1999; Studier et al., 2009). In REL4536, nine different IS element families are present, each with characteristic size, frequency and modes of transposition (Barrick et al., 2009; Mahillon & Chandler, 1998; Shewaramani et al., 2017). Of these, three were active in this LTEE: IS1, IS3 and IS150 (Fig. 4).

IS150 insertions accounted for the majority of IS-mediated mutations in lineages exposed to the anaerobic environment, and this is likely a consequence of the ∼10-fold greater spontaneous mutation rate observed for IS150 during anaerobic growth as compared to aerobic growth (Shewaramani et al., 2017). In the study by Shewaramani et al. (2017) enhanced IS150 gene expression was observed during anaerobic growth, and it was speculated that further post-transcriptional regulation of transposase expression may be affected by anaerobic physiology, though the mechanism for this is unclear. Conceivably, the accumulation of fermentation end-products, altered pH and slower growth rate may impose stresses or conditions that alter IS150 activity, and these will require further investigation. IS-mediated mutation may offer genome plasticity during adaptation to heterogeneous environments due to their transient nature and ability to insert and remove themselves from various genes within the host genome (Casacuberta & González, 2013; Gaffé et al., 2011). Movement of IS elements can affect genome structure, and also gene expression by inserting in regulatory elements (Kinnersley, Holben & Rosenzweig, 2009). Genomic hotspots for IS element insertion within REL4536 were apparent as many identical IS150 mutations were identified among different lineages, between different treatments (particularly AN and FL), and even in completely independent studies where identical IS150 mutations were observed during anaerobic mutation accumulation (Shewaramani et al., 2017). For example, IS-element mediated deletions of up to 29 genes (approximately 0.74% of genes in REL 4536) were common in the AN and FL lineages. Such mutations may have deleted non-essential DNA and resulted in a smaller genome that may allow the cell to increase their replication rate during anaerobic growth (Giovannoni et al., 2005; Lee & Marx, 2012; Mira, Ochman & Moran, 2001). The prevalence of IS150-mediated mutation among the adaptive lineages suggests such mutations are a strong driving force behind adaptation. Our observations are in contrast to a traditional view where IS elements have been considered genetic parasites that relocate and proliferate within host genomes (Charlesworth, Sniegowski & Stephan, 1994) causing potentially deleterious effects on the host cell. However, growing evidence suggests that IS element transposition has profound effects on the adaptation of organisms to their environments (Casacuberta & González, 2013; Cooper et al., 2001; Kazazian, 2004; Schneider et al., 2000; Schneider & Lenski, 2004). In this LTEE, the AN and FL lineages had the highest numbers of IS mutations, and were also those with the greatest fitness responses in the anaerobic environment.

**Figure 5:**
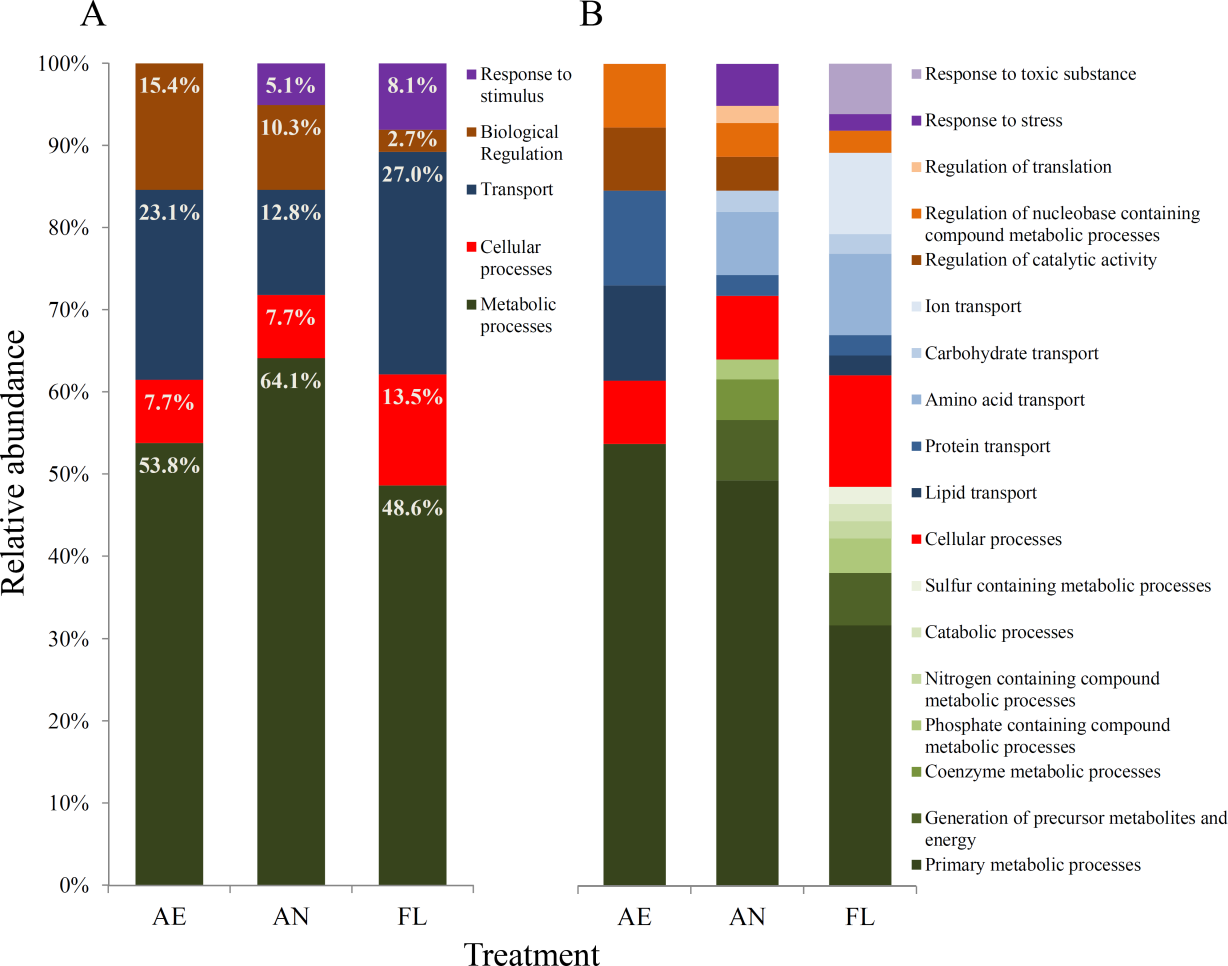
Gene ontology (GO) biological processes (A) and GO molecular functions (B) affected by mutations within genomes of evolved lineages, as defined using the PANTHER protein classification system (Mi et al., 2016).

**(ii) Effects of mutations at the biological process level** The genome ontology (GO) classification of biological processes that were impacted by mutation during evolution were similar across the three treatments (Fig. 5A).

For all three treatments, metabolic and transport processes accounted for the highest proportion of processes whose genes were impacted by mutation overall. Within the AE lineages, metabolic processes such as anabolism and catabolism, cellular processes such as the cell cycle and transport of biomolecules, and biological regulatory processes such as catalytic activities accounted for 53.8%, 7.7%, 23.1% and 15.4% of affected processes, respectively (Fig. 5). However, the relative proportions and diversity of categories at the molecular function level varied much more among AN and FL lineages than for AE (Fig. 5B).

A wider range of metabolic processes were impacted by mutation in lineages exposed to the anaerobic environment (AN and FL) than in AE lineages, where only primary metabolic processes were affected (Fig. 5B). For lineages exposed to the anaerobic environment, the diversity of transport systems impacted differed from AE lineages, where only amino acid and carbohydrate transport were affected (Fig. 5B). Furthermore, FL lineages had the highest percentage of mutations involved in transport processes (27%), where a notably large proportion was due to mutations in genes for ion transport, such as *gltP*, *btuC* and *zntB* (Table 3). Overall, the FL lineages had a greater diversity of protein functional subcategories impacted, which is consistent with the idea that the fluctuating treatment selects for mutations that underpin broad generalisation to maintain fitness across varied environments.

In contrast to the AE treatment, the AN and FL lineages had mutations in genes in response to stimulus class (Fig. 5A). In AN lineages, mutations were in genes involved in stress response, such as *appY* (Atlung, Knudsen & Heerfordt, 1997), and *envY* (Lundrigan & Earhart, 1984), while in FL lineages, additional mutations were found in mismatch repair gene, *mutL,* and genes classified in response to toxic substances, e.g., *btuE* which encodes a putative glutathione peroxidase (Fig. 5B).

**Table 3:** Adaptive mutations reported in AE, AN and FL lineages.

Predicted mechanism^a^	Category^b^	Gene/Region	Gene function^c^	Treatment^d^	Mutation	Position^e^	Lineage^f^
Altered metabolism	Fermentation pathways	*nadR*	Upregulates fermentation network *via* NAD	Anaerobic	IS150 insertion	4,581,545	AN-2K-3, 4, 5 and 6
Altered metabolism	Fermentation pathways	*adhE*	Disrupts ethanol production	Anaerobic	SNP	1,438,030	AN-2K-1, 3, 4 and 5
Altered metabolism	Fermentation pathways	*adhE*	Disrupts ethanol production	Anaerobic	SNP	1,439,673	AN-2K-2 and 7
Altered metabolism	Fermentation pathways	*dcuS*^g^	Induces succinate production	Anaerobic	Deletion	4,295,377	AN-2K-1, 2, 3, 4, 5 and 6
Altered metabolism	Fermentation pathways	*pflB*	Formate production	Anaerobic and fluctuating	IS150 deletion	1,764,888	AN-2K-2, 3 and FL-2K-1, 2, 3, 5, 6 and 7
Inactivation of redundant functions	Toxin-antitoxin system	*hokC/nhaA*	*hok/sok* system	Anaerobic and fluctuating	IS150 insertion	16,972	AN-2K-1, 4 and FL-2K-4
Inactivation of redundant functions	Toxin-antitoxin system	*hokC/nhaA*	*hok/sok* system	Anaerobic and fluctuating	IS150 insertion	16,989	FL-2K-7
Inactivation of redundant functions	Toxin-antitoxin system	*insA-7/hokE*	*hok/sok* system	Fluctuating	IS150 insertion	582,237	FL-2K-7
Inactivation of redundant functions	Toxin-antitoxin system	*trg/mokB*	*hok/sok* system	Anaerobic and fluctuating	IS150 insertion	1,272,468	AN-2K-1, 4 and FL-2K- 2, 6 and 7
Inactivation of redundant functions	Toxin-antitoxin system	*chaA/ldrC*	*ldr* system	Fluctuating	IS150 insertion	1,464,061	FL-2K-7
Inactivation of redundant functions	Toxin-antitoxin system	*ldrC/ldrB*	*ldr* system	Fluctuating	IS150 insertion	1,464,678	FL-2K-2
Inactivation of redundant functions	Toxin-antitoxin system	*ldrC/ldrB*	*ldr* system	Aerobic	IS150 insertion	1,464,679	AE-2K-1
Inactivation of redundant functions	Prophage	P22	–	Anaerobic and fluctuating	7 gene deletion	*insB-6-ompY*	FL-2K-7
Inactivation of redundant functions	Prophage	P22	–	Anaerobic and fluctuating	30 gene deletion	*insB-6-ybdK*	AN-2K-7
Inactivation of redundant functions	Prophage	Qin	–	Aerobic and fluctuating	5 gene deletion	*ydfX-ECB_01533*	FL-2K-2
Inactivation of redundant functions	Prophage	Qin	–	Aerobic and fluctuating	25 gene deletion	*ECB_01536-insE-3*	AE-2K-6
Inactivation of redundant functions	Amino acid transporters	*brnQ*	Branched chain amino acid transporter	Fluctuating	IS150 insertion	388,275	FL-2K-4 and 6
Inactivation of redundant functions	Amino acid transporters	*brnQ*	Branched chain amino acid transporter	Fluctuating	IS150 insertion	388,543	FL-2K-2
Inactivation of redundant functions	Amino acid transporters	*brnQ*	Branched chain amino acid transporter	Fluctuating	Insertion	388,020	FL-2K-3
Inactivation of redundant functions	Amino acid transporters	*brnQ*	Branched chain amino acid transporter	Fluctuating	6 gene deletion	*[araJ]-[brnQ]*	FL-2K-7
Inactivation of redundant functions	Amino acid transporters	*gltK*	Glutamate and aspartate transporter	Fluctuating	SNP	2,069,532	FL-2K-7
Inactivation of redundant functions	Amino acid transporters	*gltP*	Glutamate and aspartate transporter	Fluctuating	IS150 insertion	4,239,784	FL-2K-7
Inactivation of redundant functions	Amino acid transporters	*cycA*	Short chain amino acid permease	Anaerobic and fluctuating	IS150 insertion	4,381,583	AN-2K-1, 3, 4, 5, 6 and 7 and FL-2K-1, 2, 3, 4, 5, 6 and 7
Inactivation of redundant functions	Virulence genes	*agn43*	Outer membrane auto-transporter	Aerobic and anaerobic	SNP	2,972,858	AN-2K-7
Inactivation of redundant functions	Virulence genes	*agn43*	Outer membrane auto-transporter	Aerobic and anaerobic	SNP	2,973,574	AN-2K-3
Inactivation of redundant functions	Virulence genes	*agn43*	Outer membrane auto-transporter	Aerobic and anaerobic	IS1 insertion	2,972,936	AE-2K-5 and 7
Inactivation of redundant functions	Virulence genes	*kpsT*	Inner membrane polysaccharide transport	Aerobic	Deletion	2,999,898	AE-2K-6
Inactivation of redundant functions	Virulence genes	*kpsT*	Inner membrane polysaccharide transport	Aerobic	SNP	3,000,095	AE-2K-3 and 4
Inactivation of redundant functions	Virulence genes	*kpsT*	Inner membrane polysaccharide transport	Aerobic	SNP	3,000,161	AE-2K-1
Inactivation of redundant functions	Virulence genes	*kpsT*	Inner membrane polysaccharide transport	Aerobic	SNP	3,000,346	AE-2K-5 and 7
Inactivation of redundant functions	Virulence genes	*kpsE*	Polymer translocation	Aerobic	SNP	2,987,334	AE-2K-2
Inactivation of redundant functions	Virulence genes	*kpsD*	Polymer translocation	Aerobic	SNP	2,988,653	AE-2K-3 and 4
Inactivation of redundant functions	Virulence genes	*kpsM*	Inner membrane polysaccharide transport	Fluctuating	IS3 insertion	3,000,519	FL-2K-3
Inactivation of redundant functions	Virulence genes	*kpsS*	Post translational modification	Anaerobic	IS1 insertion	2,992,382	AN-2K-1, 3, 4, 5 and 6

**Notes.**

a The predicted mechanism of adaptation.

b The category of trait mutated.

c The function of the wild type gene.

d The treatment under which the mutations arose.

e Mutation location using REL4536 reference genome, except for prophage excision mutations which are listed as the genes in which the deletions begin and end.

f Lineages are identified by the treatment (AE, FL, or AN), followed by the generation (in all cases, approximately 2,000, designated 2K), and the individual lineage number, 1 to 7.

g Gene *dcuS* is restored by the 5 bp deletion slippage mutation.

**(iii) Parallelism between lineages** The predicted roles that mutations had on the fitness of evolving lineages was explored. Adaptive mutations in evolving lineages were inferred based on the extent of parallelism among mutations, and in consideration of the treatments under which they were identified (Table 3). Two general mechanisms of adaptation are hypothesised from these analyses: altered metabolism under the environment of selection, and the deletion or disruption of redundant functions.

### Altered metabolism

**Figure 6:**
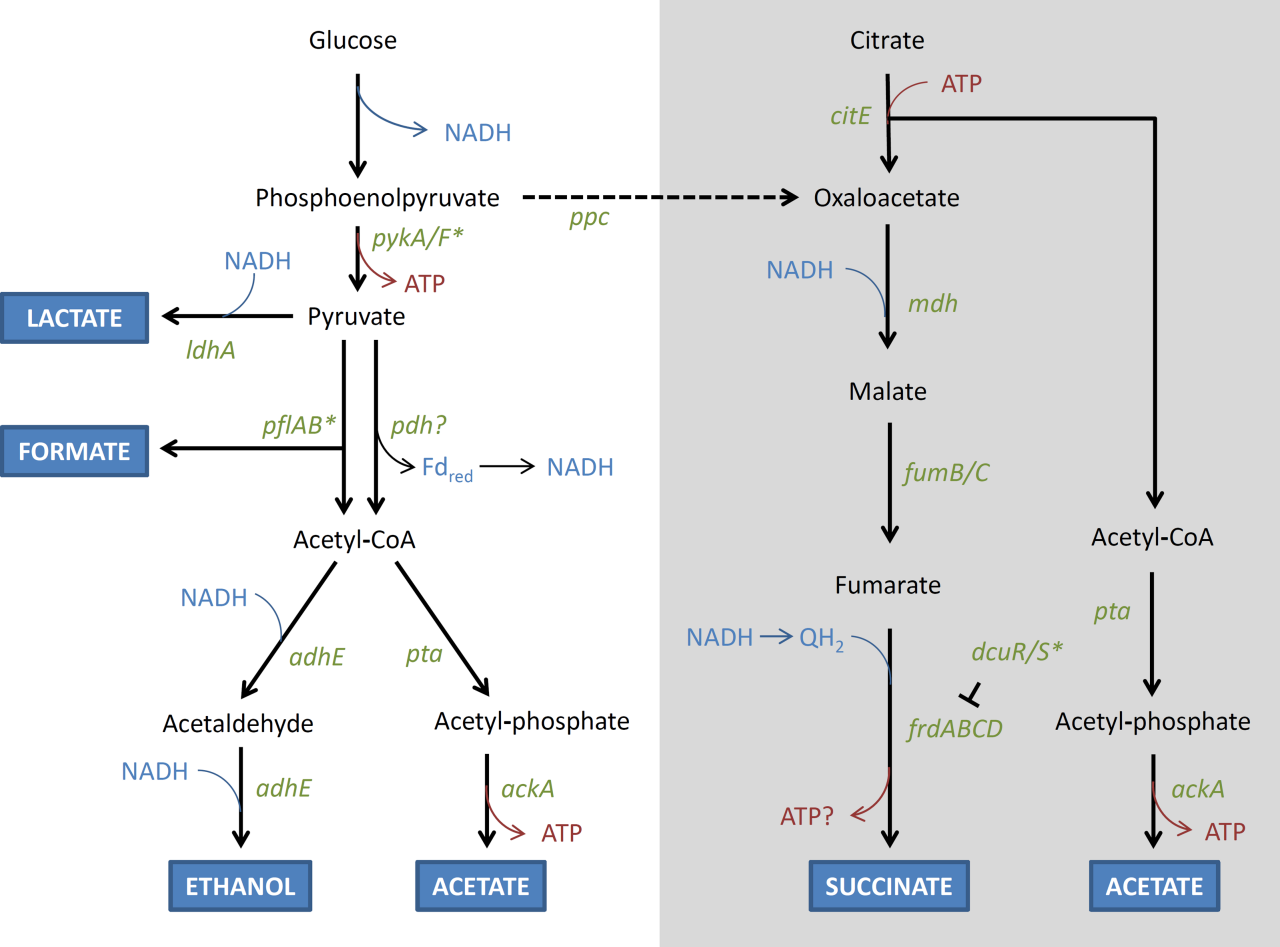
Schematic (unbalanced) diagram of the anaerobic fermentation pathways and genes involved in glucose fermentation by *E. coli*. Thick branching arrows represent cleavage into two smaller products. Where there are two thick arrows from one compound, these represent alternative fates. The grey box contains the pathway of citrate metabolism, and the dashed arrow represents the flow from glucose fermentation to the succinate pathway in mixed acid fermentation. Genes that are disrupted in REL4536 are represented by asterisks. The T symbol represents regulation, Fd_red_ represents ferredoxin, and QH_2_ represents reduced quinone.

** (a) Modification of fermentation** Mutations in genes involved in fermentation were found only among lineages that were exposed to the anaerobic environment. In particular, in both the AN and FL lineages, mutations that are predicted to (a) disrupt *nadR* and *adhE,* and (b) restore *dcuS*, and *pflB,* were identified multiple times in independent lineages (Table 3). The roles of these genes in fermentation are shown in Fig. 6. It is further noted that mutations in similar genes and functions (e.g., *adhE* and NAD^+^ metabolism) also arose when *E. coli* MC1000 was adapted to oxygen rich and oxygen poor conditions in a rich medium (Puentes-Téllez et al., 2013).

NadR is a negative transcriptional regulator of NAD biosynthetic genes, whose products catalyse the initial steps of the NAD biosynthetic pathway (San et al., 2002). Disruption of NadR is predicted to increase the intracellular concentration of NAD via the constitutive expression of *nadA* and *nadB* (Woods et al., 2006). NAD is an essential coenzyme that plays a central role in metabolism by acting as electron acceptors and donors in its oxidised (NAD^+^) and reduced (NADH) states in many biochemical redox reactions (Gerasimova & Gelfand, 2005). Fermentation pathways are highly responsive to the intracellular NADH/NAD^+^ ratio (Leonardo, Cunningham & Clark, 1993; Wimpenny & Firth, 1972). An increase in NAD concentration is likely to globally stimulate fermentation pathways under anaerobic growth conditions by increasing electron flow (Liu et al., 2006; San et al., 2002).

The REL4536 ancestral strain contains an IS150 disruption of *pflB* (Barrick et al., 2009), which encodes pyruvate formate lyase and is required for the fermentation of pyruvate to formate (Fig. 6). *E. coli* contains multiple genes coding for putative pyruvate formate lyases (Sawers & Watson, 1998), but only *pflB* seems to be involved in formate production as a catabolic product from glucose (Falke et al., 2016). Deletion of *pflB* (and not its homologue *pflD*) results in increased lactate formation (Zhu & Shimizu, 2005). Deletion of *pflB* also abolishes the ability to produce formate, acetate, and ethanol. Therefore, it is predicted that the ancestor may undertake fermentation mainly via the lactate pathway, yielding only two ATP per glucose, because it lacks the ability to produce formate as a catabolic product. In the AN-2K-2, AN-2K-3 and all FL-2K genomes, deletion of this IS150 copy is predicted to restore *pflB*, and thus allow a mixed acid fermentation that would increase ATP yield to three per glucose. Selection for this mutation under the AN and FL treatments further suggests its beneficial nature for growth in anaerobic environments.

Normally, pyruvate dehydrogenase (Pdh) is not active under anaerobic conditions as, in the absence of an electron sink, NADH and reduced ferredoxin (Fd_red_) would accumulate and metabolism would cease. However, with citrate acting as a potential electron sink, re-oxidation of NADH can take place and Pdh activity would not be limited by the build-up of reduced co-factors. Pdh is also allosterically inhibited by NADH, but this inhibition would be expected to be lower if there was an electron sink, like citrate, to ensure re-oxidation of NADH. The inhibition is also overcome by competition by NAD^+^ (Hansen & Henning, 1966), which may also explain why an active *nadR* was apparently selected for in some of the AN lineages.

Alcohol dehydrogenase (encoded by *adhE*) is required for the reduction of acetyl-CoA to ethanol in *E. coli* (Leonardo, Cunningham & Clark, 1993), and disruption of this gene appeared to be strongly selected for during anaerobic growth, with mutations detected in the majority of the AN genomes. Mutations in *adhE* may alleviate the build-up of ethanol (Zhu & Shimizu, 2005), which is toxic at high concentrations. However, they may also allow for electron flow to be diverted to other, more energetically favorable, pathways. As citrate is supplied in the medium, it may be used as an electron sink, and the citrate-succinate pathway is predicted to result in greater ATP yield (Fig. 6). However, in the ancestral strain, *dcuS* was disrupted by a frameshift caused by a 5-bp slippage event. The *dcuS* gene encodes part of the DcuR/S two-component regulatory system that regulates fumarate reductase (Zientz, Bongaerts & Unden, 1998) required for succinate formation. Restoration of *dcuS* was strongly selected for, with deletion of the 5-bp repeated motif being observed in six of the AN genomes (Table 3). The resulting reactivation of the DcuR/S system is predicted to enable use of citrate as an electron sink, by increasing fumarate reductase expression (Zientz, Bongaerts & Unden, 1998). Furthermore, the diversion of fermentation from ethanol to succinate is predicted to result in greater ATP yields that would be highly advantageous under anaerobic growth (Kwon et al., 2011).

It was considered that the production of acetate during fermentation may provide an alternative carbon source for lineages exposed to the anaerobic environment of this LTEE as *E. coli* is capable of metabolising acetate as an alternative to glucose (Le Gac et al., 2008; Rosenzweig et al., 1994). Acetate metabolism among evolving *E. coli* experimental lineages has been found in other LTEE studies performed in aerobic environments and is hypothesised to mediate a cross-feeding dynamic among co-evolving populations (Treves, Manning & Adams, 1998). However, this is unlikely to be advantageous under strictly anaerobic growth conditions due to lack of an electron acceptor.

It is noted that the inactivation of both *dcuS* and *pflB* in the REL4536 ancestor was likely to have had little impact during the long-term aerobic selection from which it had derived (Barrick et al., 2009). However, in the present LTEE, the functions of both genes were restored in lineages exposed to the anaerobic environment, suggesting that they are important for adaptation in this genetic background.

The current study has highlighted that many mutations that arose in the Lenski LTEE impacted genes that are beneficial for anaerobic growth, such as *pflB, pflC*, *pykF*, *arcB* and *nadR* (Barrick et al., 2009). In Lenski’s study, the low glucose concentration in the growth medium gives rise to low cell densities, thus oxygen availability is not likely limiting for growth. This is in contrast to typical shaken batch broth cultures, where anaerobicity is typically reached and a switch to fermentation is required for further growth (Vasala et al., 2006). Furthermore, end products are unlikely to accumulate to inhibitory levels in such low density cultures. As such, the disruption of genes involved in anaerobic growth was unlikely to negatively impact fitness during selection in Lenski’s LTEE (Barrick et al., 2009). In the current study, the use of a 10,000th generation evolved clone from Lenski’s LTEE was primarily to overcome adaptation to the growth medium. However, anaerobic adaptation has selected for mutations that reverse the effects of mutations that arose under prolonged aerobic selection in Lenski’s LTEE. Overall, the mutations in genes affecting fermentation pathways identified in this study are likely to alter the metabolic activity of the organism by changing its ability to dispose of electrons, and in doing so, improve the efficiency of energy production during anaerobic growth. Testing the impacts of these mutations and pathways on fermentation and energy production efficiency will be the subject of ongoing research.

### Disruption of redundant functions

The second general mechanism of adaptation observed in this LTEE involved mutations that are predicted to eliminate physiological functions that are likely not to be required in the environment of selection. Indeed, this seems to have happened in the evolution of the REL4536 ancestor strain used here, as described above. The majority of the new mutations in this study did not exclusively occur in one treatment or environment, and so it is likely that these contribute to adaption via conditions that were common between the treatments, such as growth media. Furthermore, these mutations likely accounted for a comparatively lower fitness response than the mutations that altered metabolic function, as they were present in AE lineages, which did not increase in fitness to the same extent as the AN or FL lineages. The functions affected by such mutations included inactivation of toxin-antitoxin (TA) systems, prophage excisions, inactivation of certain amino acid transporters, and inactivation of virulence associated genes.

**(a) Inactivation of TA systems** Multiple independent mutations were found in the intergenic regions adjacent to *hok*/*sok* and *ldr* TA system genes, predominantly in the FL genomes, but also in some AE and AN clones (Table 3). TA systems are widely distributed among prokaryotes where, typically, they are located on plasmids and involved in post segregational killing of plasmid-free daughter cells (Gerdes & Wagner, 2007). A large proportion of the non-coding DNA mutations identified (Fig. 3) were in the cis-regulatory regions of TA system genes, such as the upstream complementary box (*ubc*), or translational activator element (*tae*) (Pedersen, Christensen & Gerdes, 2002). Plasmid maintenance may be advantageous when plasmids encode beneficial functions such as antibiotic resistance. However, the ancestral strain in this study did not contain plasmids, thus post segregational killing is presumed to be redundant in this context. Alternatively, it has been proposed that post segregational killing may no longer be the primary role of these TA systems (Mruk & Kobayashi, 2014) and that chromosomal TA systems may function in cell cycle arrest or programmed cell death, triggered under stressful conditions, such as oxygen limitation (Hayes, 2003; Mruk & Kobayashi, 2014). Disruption of TA gene function may be a strategy to ensure programmed cell death is forgone in favour of adaptation during exposure to the anaerobic environment.

**(b) Prophage excisions** Prophages are often maintained in bacterial genomes because they may confer beneficial phenotypes such as resistance to antibiotics and increased oxidative stress tolerance (Osman et al., 2010; Wang et al., 2010; Wang, Kim & Wood, 2009). However, prophages can encode activities that can cause profound metabolic changes in the cell (Edlin, Lin & Bitner, 1977). Prophage excisions accounted for 36% of the large deletions reported in this LTEE, and were responsible for 75% of genes deleted. Specifically, deletions impacted cryptic prophages, or the remnants of prophages Qin and P22. Qin and P22 sequences account for 47 kb, or ∼1%, of the REL4536 genome, thus their deletions may be selected in order to lower the metabolic burden on the cell during replication.

**(c) Inactivation of amino acid transporters** Mutations were found in genes *brnQ*, *gltK*, *gltP* and *cycA,* which encode various amino acid transporters, in lineages that evolved in the anaerobic environment (Table 3). In particular, a high level of parallelism was observed in *brnQ* among FL lineages (Table 3), which encodes a symporter for the uptake of LIV family branched chain amino acids (Table 3) (Saier Jr, 2000; Stucky et al., 1995). The *glt* genes encode one of four different transporters for the uptake of glutamate and aspartate in *E. coli* (Schellenberg & Furlong, 1977). Strong selection for the IS150 disruption of *cycA* under anaerobic conditions occurred in 13 AN and FL lineages (Table 3). This gene encodes a transporter involved in the uptake of glycine, d-serine and d-alanine (Baisa, Stabo & Welch, 2013). Among the three treatments, FL lineages had the highest proportion of deleterious mutations in amino acid uptake systems in an environment where free amino acids are limited, and may be a mechanism of economising the cell’s resources, allowing it to better cope with starvation and/or fluctuating conditions (D’Souza et al., 2015).

**Figure 7:**
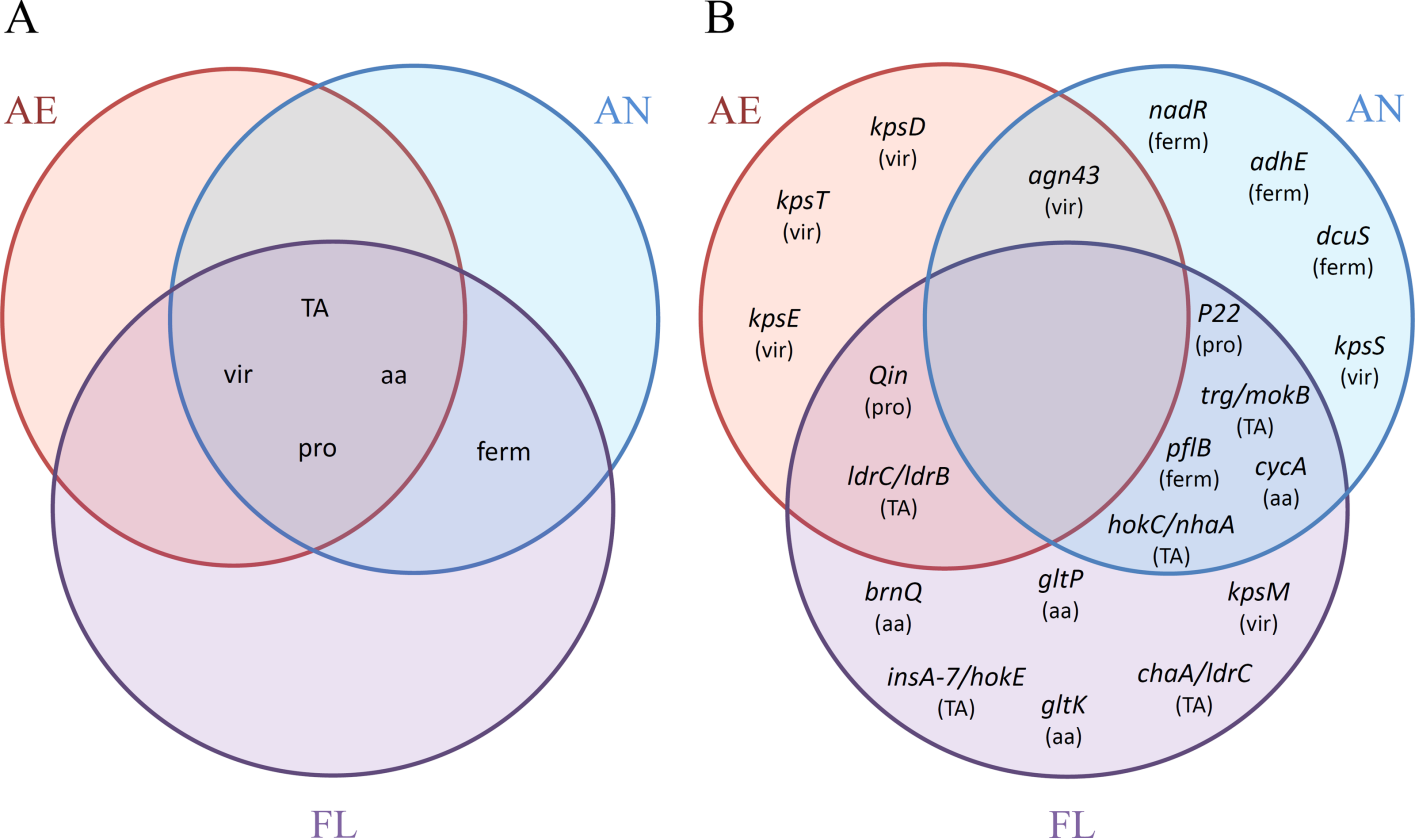
Venn diagrams of the distributions of (A) categories (TA, TA systems; vir, virulence genes; aa, amino acid transporters; pro, prophage excision; ferm, fermentation network) and (B) adaptive mutations, with corresponding categories affected in parenthesis, as reported among the AE, AN and FL genomes after 2,000 generations.

**(d) Inactivation of virulence genes** Numerous mutations were identified in genes associated with virulence activity in *E. coli*, specifically in *agn43* (previously known as *flu*) and the *kps* operon (Table 3). Antigen 43 (Ag43) is an outer membrane auto-transporter, and is a virulence factor associated with infection in pathogenic strains of *E. coli* (Van der Woude & Henderson, 2008). Mutations were predicted to disrupt the α domain of Ag43 which confers the auto-aggregation phenotype of the virulent protein (Van der Woude & Henderson, 2008). The *kps* operon is involved in the assembly of an extracellular capsid found in pathogenic strains of *E. coli* (Scholl, Adhya & Merril, 2005; Schwan et al., 2005). This feature is likely to be advantageous in the wild, but not under the conditions of this LTEE.

### Adaptation to aerobic and anaerobic environments

A summary of the adaptive traits and impacted genes affected by mutation in the LTEE for each treatment group is shown in Fig. 7. None of the general traits impacted were found solely within a single treatment group (Fig. 7A), but were either shared among all three treatments (inactivation of virulence genes, TA systems and prophage excisions), or shared between treatments that were exposed to the anaerobic environment (i.e., the AN and FL lineages only). However, upon examination of specific genes within each category, mutations in particular genes appeared to have greater treatment specificity (Fig. 7B). For example, mutations to *dcuS* were only found in the AN evolved lineages, and *hok*/*sok* TA system-based disruptions were only found in lineages exposed to anaerobic conditions, whereas the *ldr* TA system mutations were only found in lineages exposed to the aerobic environment. Whether these are environment-specific adaptations requires further investigation.

## Conclusions

We examined the experimental evolution of *E. coli* REL4536 in aerobic and anaerobic environments with constant and non-constant exposure. Selection in the strictly anaerobic environment resulted in fitness increases of up to 40% over 2,000 generations of selection.

Mutations that had arisen during the 2,000 generations of evolution were identified, where IS150 element-mediated mutations in non-coding regions and gene disruptions played a large role in the anaerobically-grown cells. Biological processes impacted by mutation were relatively similar among treatments, though metabolic and transport processes were affected more in the AN and FL lineages, respectively. Adaptation to the anaerobic environment was mediated by mutations that were predicted to improve energy yield during fermentation and eliminate functions redundant to the anaerobic environment. Our results demonstrate the dynamic nature of genomes in response to natural selection in the anaerobic environment and reinforce the importance of studying diverse environments to enhance our understanding of evolution in the wild.

##  Supplemental Information

10.7717/peerj.3244/supp-1 Supplemental Information 1 Supplementary Tables

## Additional Information and Declarations

### Data Availability

The following information was supplied regarding data availability: The raw sequence data generated in this study are accessible via the GenBank BioProject ID PRJNA357967.
