# Supplementary material for: Dynamics and genetic diversification of Escherichia coli during experimental adaptation to an anaerobic environment
Source: PeerJ. 2017 May 3;5:e3244. doi: 10.7717/peerj.3244 (PMC5419217; doi:10.7717/peerj.3244)
Supplement: Supplemental Information 1 [file peerj-05-3244-s001.docx]

Supplementary Table 1 List of all mutations in AE lineages

| Lineage | Mutation  class | Mutation  sub-class^a^ | Reference genome position 1^b^ | Size (bp) | Base change | Amino  acid change | Functional effect^c^ | Reference Gene 1^d^ | Gene 1 description | Reference  Gene 2^e^ | Gene 2 description | General comment |
| --- | --- | --- | --- | --- | --- | --- | --- | --- | --- | --- | --- | --- |
| 1 | SNP | Transversion | 700,702 | 1 | G→T | Gly (48) → Val | non-synonymous | *gnd* | 6‑phosphogluconate dehydrogenase |  |  |  |
|  | Mobile element | Insertion | 1,464,679 | 1,471 |  |  | non-coding | *ldrC*  (+344) | *ldrC* is one of a set of four Long Direct Repeats (LDRs), one of which has been shown to code for the toxin portion of a toxin-antitoxin pair. | *ldrB*  (-84) | *ldrB* is one of a set of four Long Direct Repeats (LDRs), one of which has been shown to code for the toxin portion of a toxin-antitoxin pair. | IS150 mediated, intergenic |
|  | SNP | Transversion | 1,943,095 | 1 | A→T | Ile (355) → Thr | non-synonymous | *ECB_00733* | Hypothetical protein |  |  |  |
|  | SNP | Transition | 3,000,161 | 1 | T→C | Tyr (111) → Cys | non-synonymous | *kpsT* | Polysialic acid transport ATP binding protein KpsT |  |  |  |
|  | Deletion | Small | 3,866,358 | 1 | -G |  | non-coding | *trkD*  (+6) | Potassium transport protein Kup | *insJ-5*  (-51) | IS150 putative transposase | Intergenic |
|  | Deletion | Small | 3,524,253 - 3,525,072 | 173 - 1,048 |  |  | indel frameshift | *rhsB* | rhsB element core protein RshB |  |  |  |
| 2 | SNP | Transition | 109,384 | 1 | A→G | Lys (8) → Lys | synonymous | *ipxC* | N-acetylglucosamine deacetylase |  |  |  |
|  | SNP | Transition | 2,052,242 | 1 | G→A | Gly (308) →Ser | non-synonymous | *nagA* | N-acetylglucosamine -6- phosphate deacetylase |  |  |  |
|  | SNP | Transversion | 2,987,334 | 1 | A→C | Thr (365) → Pro | non-synonymous | *kpsE* | Capsule polysaccharide export protein KpsE |  |  |  |
|  | SNP | Transition | 4,217,932 | 1 | G→A | Ala (249) → Thr | non-synonymous | *uvrA* | UvrABC DNA lesion repair protein |  |  |  |
| 3 | SNP | Transversion | 2,988,653 | 1 | A→T | Ile (418) → Phe | non-synonymous | *kpsD* | Polysialic acid transporter |  |  |  |
|  | SNP | Transition | 3,000,095 | 1 | T→C | Met (133) → Thr | non-synonymous | *kpsT* | Polysialic acid transport ATP binding protein KpsT |  |  |  |
|  | Deletion | Small | 3,915,605 | 6 |  |  | indel in frame | *hemX* | Putative uroporphyrinogen III C- methyltransferase |  |  |  |
|  | SNP | Transversion | 4,107,509 | 1 | A→C | Lys  (2) → Thr | non-synonymous | *yijD* | Hypothetical protein |  |  |  |
| 4 | SNP | Transition | 323,923 | 1 | T→C | Val (20) → Ala | non-synonymous | *prpD* | 2- methylcitrate dehydratase |  |  |  |
|  | SNP | Transversion | 2,988,653 | 1 | A→T | Ile (418) → Phe | non-synonymous | *kpsD* | Polysialic acid transporter |  |  |  |
|  | SNP | Transition | 3,000,095 | 1 | A→G | Met (133) → Thr | non-synonymous | *kpsT* | Polysialic acid transport ATP binding protein KpsT |  |  |  |
| 5 | SNP | Transversion | 863,948 | 1 | T→G | Ile (148) → Met | non-synonymous | *fadD* | Long chain fatty acid CoA ligase |  |  |  |
|  | Deletion | Small | 892,201 | 1 |  |  | indel frameshift | *yeaA* | Methionine sulfoxide reductase B |  |  |  |
|  | Mobile element | Insertion | 2,972,936 | 777 |  |  | gene disruption | *agn43* | Antigen 43 (Ag43) phase-variable biofilm formation autotransporter |  |  | IS1 mediated |
|  | SNP | Transversion | 3,000,346 | 1 | G→C | Asp (133) → Thr | non-synonymous | *kpsT* | Polysialic acid transport ATP binding protein KpsT |  |  |  |
|  | Insertion |  | 3,866,357 | 1 | +G |  | non-coding | *trkD* (+5) | Potassium transport protein Kup | *insJ-5*  (-52) | IS150 putative transposase | Intergenic |
| 6 | SNP | Transition | 92,892 | 1 | T→C | Leu (152) → Pro | non-synonymous | *yabB* | Cell division protein MraZ |  |  |  |
|  | Deletion | Large | 1,111,336 | 12,764 - 13,978 | 25 genes |  |  | *[ECB_01536]* | 25 genes involved in 8 unknown, | *insE-3* |  | IS3 mediated |
|  | Deletion | Small | 2,999,898 | 1 |  |  | indel frameshift | *kpsT* | Polysialic acid transport ATP binding protein KpsT |  |  | Likely slippage |
|  | SNP | Transversion | 3,119,520 | 1 | C→A | Arg (279) → Ser | non-synonymous | *rpoD* | RNA polymerase sigma factor RpoD |  |  |  |
| 7 | SNP | Transversion | 863,948 | 1 | T→G | Ile (148) → Met | non-synonymous | *fadD* | Long chain fatty acid CoA ligase |  |  |  |
|  | Deletion | Small | 892,201 | 1 |  |  | indel frameshift | *yeaA* | Methionine sulfoxide reductase B |  |  |  |
|  | Mobile element | Insertion | 2,972,936 | 777 |  |  | gene disruption | *agn43* | Antigen 43 (Ag43) phase-variable biofilm formation autotransporter |  |  | IS1 mediated |
|  | SNP | Transition | 3,000,346 | 1 | G→C | Asp (133) → Thr | non-synonymous | *kpsT* | Polysialic acid transport ATP binding protein KpsT |  |  |  |
|  | Insertion | Small | 3,866,357 | 1 | +G |  | non-coding | *trkD*  (+5) | Potassium transport protein Kup | *insJ-5*  (-52) | IS150 putative transposase | Intergenic |

^a^ Sub-classification of mutation: SNP (transversion, transition), mobile element (insertion, deletion), deletion (small <10 bp, large >11 bp), insertion (small <10, duplication).

^b^ The position on the reference genome at which the mutation occurred. In some cases, particularly with large deletions, the exact location of the beginning of the mutation could not be specified from the breseq output, and instead, an indicative size range is given.

^c^ Frameshifts, gene disruptions and non-synonymous mutations are predicted to change protein function.

^d^ The mutated gene. In the case of genes partially deleted as part of a large multi-gene deletion, these are shown in parentheses [ ]. Intergenic mutations are identified by the gene closest to the mutation, with distance from the beginning of the gene (“+” for downstream and “-“ for upstream) shown in bp.

^e^ For intergenic mutations, the nearest gene downstream of the mutation, Reference Gene 2, is listed, with the distance (in bp) from Reference Gene 2 shown in parentheses.

Supplementary Table 2 List of all mutations in AN lineages

| Lineage | Mutation  class | Mutation  sub-class^a^ | Reference genome position 1^b^ | Size (bp) | Base change | Amino  acid change | Functional effect^c^ | Reference Gene 1^d^ | Gene 1 description | Reference  Gene 2^e^ | Gene 2 description | General comment |
| --- | --- | --- | --- | --- | --- | --- | --- | --- | --- | --- | --- | --- |
| 1 | Deletion | Large | 998,264 -999,628 | 2,647 - 4,011 | 4 genes |  |  | *[pykF]* | Pyruvate kinase I | *[ydhV]* | Oxidoreductase system | IS150 mediated insertion in *pykF* and *ydhV* |
|  | Mobile element | Insertion | 621,252 | 1,474 |  |  | gene disruption | *citG* | Triphosphoriboseyl-dephospho-CoA synthase |  |  | IS150 mediated |
|  | Mobile element | Insertion | 872,829 | 1,446 |  |  | gene disruption | *yeaS* | Leucine export protein LeuE |  |  | IS150 mediated |
|  | Mobile element | Insertion | 910,345 | 1,446 |  |  | gene disruption | *ynjI* | Hypothetical protein |  |  | IS150 mediated |
|  | Mobile element | Insertion | 2,387,885 | 1,264 |  |  | gene disruption | *emrY* | Putative multidrug efflux system |  |  | IS3 mediated |
|  | Mobile element | Insertion | 2,564,470 | 1,446 |  |  | gene disruption | *hcaC* | 3-phenylpropionate dioxygenase ferredoxin subunit |  |  | IS150 mediated |
|  | Mobile element | Insertion | 2,992,382 | 777 |  |  | gene disruption | *kpsS* | KpsS protein capsular biosynthesis |  |  | IS1 mediated |
|  | Mobile element | Insertion | 3,096,895 | 777 |  |  | gene disruption | *yqiI* | Hypothetical protein |  |  | IS1 mediated |
|  | Mobile element | Insertion | 4,381,583 | 1,472 |  |  | gene disruption | *cycA* | D-alanine, D-serine, glycine permease |  |  | IS150 mediated |
|  | Mobile element | Insertion | 4,401,824 | 1,446 |  |  | gene disruption | *ytfT* | Putative sugar transporter subunit membrane component of ABC superfamily |  |  | IS150 mediated |
|  | Mobile element | Insertion | 4,581,545 | 1,446 |  |  | gene disruption | *nadR* | Nicotinamide‑nucleotide adenylyltransferase |  |  | IS150 mediated |
|  | Deletion | Small | 4,295,377 | 5 |  |  | indel frameshift | *dcuR*  (-755) | DNA binding transcriptional activator DcuR | *yjdI*  (-1,055) | Hypothetical protein | Intergenic |
|  | Mobile element | Insertion | 16,972 | 1,446 |  |  | non-coding | *hokC*  (-71) | Small toxic membrane polypeptide | *nhaA*  (-514) | pH dependent sodium or proton antiporter | IS150 mediated, intergenic |
|  | Mobile element | Insertion | 1,272,468 | 1,446 |  |  | non-coding | *trg*  (-326) | Methyl accepting chemotaxis protein III | *mokB*  (-13) | Ribose and galactose sensor receptor and regulatory peptide | IS150 mediated, intergenic |
|  | SNP | Transition | 1,238,659 | 1 | C→T | Gln (11) → Stop | non-synonymous | *ansP* | L-asparagine transporter |  |  |  |
|  | SNP | Transition | 1,439,030 | 1 | A→G | Tyr (75) → Cys | non-synonymous | *adhE* | Alcohol dehydrogenase |  |  |  |
| 2 | Mobile element | Insertion | 227,515 | 1,446 |  |  | gene disruption | *rrsH* | 16S ribosomal RNA |  |  | IS150 mediated |
|  | Mobile element | Insertion | 494,056 | 1,446 |  |  | gene disruption | *ybbP* | Putative inner membrane protein |  |  | IS150 mediated |
|  | Mobile element | Insertion | 2,024,212 | 1,446 |  |  | gene disruption | *rhsC* | rhsC element core protein RshC |  |  | IS150 mediated |
|  | Mobile element | Deletion | 1,764,886 | 1,446 |  |  | gene restoration | *pflB* | Pyruvate formate lyase 1 |  |  | IS150 mediated |
|  | Deletion | Small | 4,295,377 | 5 |  |  | indel frameshift | *dcuR*  (-755) | DNA binding transcriptional activator DcuR | *yjdI*  (-1,055) | Hypothetical protein | Intergenic |
|  | Mobile element | Insertion | 3,994,146 | 1,446 |  |  | non-coding | *polA*  (+223) | DNA polymerase I | *engB*  (+156) | Ribosome biogenesis GTP-binding protein YsxC | IS150 mediated |
|  | SNP | Transition | 1,439,673 | 1 | G→A | Ala (623) → Thr | non-synonymous | *adhE* | Alcohol dehydrogenase |  |  |  |
|  | Deletion | Large | 2,235,631 -2,236,332 | 9,655 - 10,356 | 9 genes |  | partial gene deletion | [*insB‑17*] | After frameshift event,transcribed with InsA mediates IS1 transposition | *[atoB]* | Short chain carbon (C4 to C6) catabolism | IS1 mediated |
|  | SNP | Transversion | 2,844,146 | 1 | T→A | Ala (35) → Ala | synonymous | *galR* | DNA binding transcriptional regulator GalR |  |  |  |
| 3 | Mobile element | Insertion | 621,252 | 1,474 |  |  | gene disruption | *citG* | Triphosphoribosyl-dephospho-CoA synthase |  |  | IS150 mediated |
|  | Mobile element | Insertion | 1,071,398 | 1,446 |  |  | gene disruption | *tus* | DNA replication terminus site-binding protein |  |  | IS150 mediated |
|  | Mobile element | Insertion | 2,564,470 | 1,446 |  |  | gene disruption | *hcaC* | 3-phenylpropionate dioxygenase ferredoxin subunit |  |  | IS150 mediated |
|  | Mobile element | Insertion | 2,850,178 | 1,446 |  |  | gene disruption | *kduD* | 2-deoxy-D-gluconate 3-dehydrogenase |  |  | S150 mediated |
|  | Mobile element | Insertion | 2,992,382 | 777 |  |  | gene disruption | *kpsS* | KpsS protein capsular biosynthesis |  |  | IS1 mediated |
|  | Mobile element | Insertion | 3,367,380 | 1,264 |  |  | gene disruption | *bfr* | Bacterioferritin |  |  | IS3 mediated |
|  | Mobile element | Insertion | 3,808,730 | 1,446 |  |  | gene disruption | *yidX* | Hypothetical protein |  |  | S150 mediated |
|  | Mobile element | Insertion | 4,381,583 | 1,472 |  |  | gene disruption | *cycA* | D-alanine, D-serine, glycine permease |  |  | IS150 mediated |
|  | Mobile element | Insertion | 4,581,545 | 1,446 |  |  | gene disruption | *nadR* | Nicotinamide-nucleotide adenylyltransferase |  |  | IS150 mediated |
|  | Mobile element | Deletion | 1,764,886 | 1,446 |  |  | gene restoration | *pflB* | Pyruvate formate lyase 1 |  |  | IS150 mediated |
|  | Deletion | Small | 4,295,377 | 5 |  |  | indel frameshift | *dcuR*  (-755) | DNA binding transcriptional activator DcuR | *yjdI*  (-1,055) | Hypothetical protein | Intergenic |
|  | SNP | Transition | 161,770 | 1 | G→A | Arg (59) → Cys | non-synonymous | *pcnB* | Poly(A) polymerase I |  |  |  |
|  | SNP | Transition | 1,438,030 | 1 | A→G | Tyr (75) →Cys | non-synonymous | *adhE* | Alcohol dehydrogenase |  |  |  |
|  | SNP | Transversion | 2,973,574 | 1 | G→T | Gly (397) →Trp | non-synonymous | *agn43* | Antigen 43 (Ag43) phase‑variable biofilm formation autotransporter |  |  |  |
| 4 | Mobile element | Insertion | 621,252 | 1,474 |  |  | gene disruption | *citG* | Triphosphoriboseyl-dephospho-CoA synthase |  |  | IS150 mediated |
|  | Mobile element | Insertion | 872,829 | 1,446 |  |  | gene disruption | *yeaS* | Leucine export protein LeuE |  |  | IS150 mediated |
|  | Mobile element | Insertion | 910,345 | 1,446 |  |  | gene disruption | *ynjI* | Hypothetical protein |  |  | IS150 mediated |
|  | Mobile element | Insertion | 2,387,885 | 1,264 |  |  | gene disruption | *emrY* | Putative multidrug efflux system |  |  | IS3 mediated |
|  | Mobile element | Insertion | 2,564,470 | 1,446 |  |  | gene disruption | *hcaC* | 3-phenylpropionate dioxygenase ferredoxin subunit |  |  | IS150 mediated |
|  | Mobile element | Insertion | 2,992,382 | 777 |  |  | gene disruption | *kpsS* | KpsS protein capsular biosynthesis |  |  | IS1 mediated |
|  | Mobile element | Insertion | 3,096,895 | 777 |  |  | gene disruption | *yqiI* | Hypothetical protein |  |  | IS1 mediated |
|  | Mobile element | Insertion | 4,381,583 | 1,472 |  |  | gene disruption | *cycA* | D-alanine, D-serine, glycine permease |  |  | IS150 mediated |
|  | Mobile element | Insertion | 4,401,824 | 1,446 |  |  | gene disruption | *ytfT* | Putative sugar transporter subunit membrane component of ABC superfamily |  |  | IS150 mediated |
|  | Mobile element | Insertion | 4,581,545 | 1,446 |  |  | gene disruption | *nadR* | Nicotinamide‑nucleotide adenylyltransferase |  |  | IS150 mediated |
|  | Deletion | Small | 4,295,377 | 5 |  |  | indel frameshift | *dcuR*  (-755) | DNA binding transcriptional activator DcuR | *yjdI*  (-1,055) | Hypothetical protein | Intergenic |
|  | Mobile element | Insertion | 16,972 | 1,446 |  |  | non-coding | *hokC*  (-71) | Small toxic membrane polypeptide | *nhaA*  (-514) | pH dependent sodium or proton antiporter | IS150 mediated, intergenic |
|  | Mobile element | Insertion | 1,272,468 | 1,446 |  |  | non-coding | *trg*  (-326) | Methyl accepting chemotaxis protein III | *mokB*  (-13) | Ribose and galactose sensor receptor and regulatory peptide | IS150 mediated, intergenic |
|  | SNP | Transition | 1,238,659 | 1 | C→T | Gln (11) →Stop | non-synonymous | *ansP* | L-asparagine transporter |  |  |  |
|  | SNP | Transition | 1,439,030 | 1 | A→G | Tyr (75) →Cys | non-synonymous | *adhE* | Alcohol dehydrogenase |  |  |  |
| 5 | Mobile element | Insertion | 910,345 | 1,446 |  |  | gene disruption | *ynjI* | Hypothetical protein |  |  | IS150 mediated |
|  | Mobile element | Insertion | 955,693 | 1,446 |  |  | gene disruption | *pheS* | Phenylalanyk -tRNA synthesis subunit alpha |  |  | IS150 mediated |
|  | Mobile element | Insertion | 2,164,019 | 1,473 |  |  | gene disruption | *mglB* | Methyl-galactoside transporter subunit |  |  | IS150 mediated |
|  | Mobile element | Insertion | 2,980,273 | 1,446 |  |  | gene disruption | *ECB_02804* | Hypothetical protein |  |  | IS150 mediated |
|  | Mobile element | Insertion | 2,992,382 | 777 |  |  | gene disruption | *kpsS* | KpsS protein capsular biosynthesis |  |  | IS1 mediated |
|  | Mobile element | Insertion | 3,495,511 | 1,446 |  |  | gene disruption | *ugpB* | Glycerol-3-phosphate transporter periplasmic binding protein |  |  | IS150 mediated |
|  | Mobile element | Insertion | 4,381,583 | 1,472 |  |  | gene disruption | *cycA* | D-alanine, D-serine, glycine permease |  |  | IS150 mediated |
|  | Mobile element | Insertion | 4,581,545 | 1,446 |  |  | gene disruption | *nadR* | Nicotinamide-nucleotide adenylyltransferase |  |  | IS150 mediated |
|  | Insertion | Duplication | 2,603,060 | 19 | ×2 |  | indel frameshift | *[rseB]* | Anti-sigma factor, negative regulator of sigma E | *[rseA]* | Anti-sigma factor, negative regulator of sigma E |  |
|  | Deletion | Small | 4,295,377 | 5 |  |  | indel frameshift | *dcuR*  (-755) | DNA binding transcriptional activator DcuR | *yjdI*  (-1,055) | Hypothetical protein | Intergenic |
|  | Deletion | Small | 1,766,329 | 3 |  |  | indel in frame | *pflB* | Pyruvate formate lyase I |  |  |  |
|  | Insertion |  | 3,866,357 | 1 | +G |  | non-coding | *trkD* (+5) | Potassium transport protein Kup | *insJ-5*  (-52) | IS150 putative transposase | Intergenic |
|  | Mobile element | Insertion | 4,211,817 | 1,446 |  |  | non-coding | *alr (+151)* | Alanine racemase | *tyrB*  (-102) | Aromatic amino acid aminotrasferase | IS150 mediated, intergenic |
|  | SNP | Transition | 1,438,030 | 1 | A→G | Tyr (75) → Cys | non-synonymous | *adhE* | Alcohol dehydrogenase |  |  |  |
|  | SNP | Transition | 2,001,307 | 1 | G→A | Val (134) → Ile | non-synonymous | *gltA* | Type II citrate synthase |  |  |  |
| 6 | Mobile element | Insertion | 910,345 | 1,446 |  |  | gene disruption | *ynjI* | Hypothetical protein |  |  | IS150 mediated |
|  | Mobile element | Insertion | 2,164,019 | 1,473 |  |  | gene disruption | *mglB* | Methyl-galactoside transporter subunit |  |  |  |
|  | Mobile element | Insertion | 2,980,273 | 1,446 |  |  | gene disruption | *ECB_02804* | Hypothetical protein |  |  | IS150 mediated |
|  | Mobile element | Insertion | 2,992,382 | 777 |  |  | gene disruption | *kpsS* | KpsS protein capsular biosynthesis |  |  | IS1 mediated |
|  | Mobile element | Insertion | 3,495,511 | 1,446 |  |  | gene disruption | *ugpB* | Glycerol-3-phosphate transporter periplasmic binding protein |  |  | IS150 mediated |
|  | Mobile element | Insertion | 4,381,583 | 1,472 |  |  | gene disruption | *cycA* | D-alanine, D-serine, glycine permease |  |  | IS150 mediated |
|  | Mobile element | Insertion | 4,581,545 | 1,446 |  |  | gene disruption | *nadR* | Nicotinamide-nucleotide adenylyltransferase |  |  | IS150 mediated |
|  | Insertion | Duplication | 2,603,060 | 19 | ×2 |  | indel frameshift | *[rseB]* | Anti-sigma factor, negative regulator of sigma E | *[rseA]* | Anti-sigma factor, negative regulator of sigma E |  |
|  | Deletion | Small | 4,295,377 | 5 |  |  | indel frameshift | *dcuR*  (-755) | DNA binding transcriptional activator DcuR | *yjdI*  (-1,055) | Hypothetical protein | Intergenic |
|  | Insertion | Small | 3,866,357 | 1 | +G |  | non-coding | *trkD* (+5) | Potassium transport protein Kup/ | *insJ-5*  (-52) | IS150 putative transposase | Intergenic |
|  | Mobile element | Insertion | 4,211,817 | 1,446 |  |  | non-coding | *alr* (+151) | Alanine racemase | *tyrB*  (-102) | Aromatic amino acid aminotrasferase | IS150 mediated, intergenic |
| 7 | Deletion | Large | 546,986 – 547,702 | 32,570 - 33,286 | 29 genes |  |  | *insB-6* |  | *ybdK* |  | IS150 mediated |
|  | Mobile element | Insertion | 1,123,058 | 1,264 |  |  | gene disruption | *ynfN* | Hypothetical protein |  |  | IS3 mediated |
|  | Mobile element | Insertion | 4,381,583 | 1,472 |  |  | gene disruption | *cycA* | D-alanine, D-serine, glycine permease |  |  | IS150 mediated |
|  | Deletion | Small | 161,094 | 2 |  |  | indel frameshift | *pcnB* | Poly(A) polymerase I |  |  |  |
|  | Insertion |  | 3,298,183 | 1 | +C |  | indel frameshift | *rng* | Ribonuclease G |  |  |  |
|  | Deletion | Small | 3,473,571 | 1 | -C |  | indel frameshift | *glgC* | Glucose-1-phosphate adenylyltransferase |  |  |  |
|  | Insertion | Small | 4,406,085 | 7 | +GTGGCAG |  | indel frameshift | *mpl* | Murein recycling and cell wall remodelling |  |  |  |
|  | Deletion | Small | 3,260,806 | 42 |  |  | indel in frame | *arcB* | Aerobic respiration control sensor protein ArcB |  |  |  |
|  | Insertion | Small | 3,866,357 | 1 | +G |  | non-coding | *trkD* (+5) | Potassium transport protein Kup | *insJ-5*  (-52) | IS150 putative transposase | Intergenic |
|  | Mobile element | Insertion | 2,424,083 | 1,472 |  |  | non-coding | *alaW*  (-154) | tRNA-Ala | *yfeC*  (-82) | Putative DNA-binding transcriptional activator | IS150 mediated |
|  | Insertion | Small | 3,866,357 | 1 | +G |  | non-coding | *trkD (+5)* | Potassium transport protein Kup | *insJ-5*  (-52)- |  | IS150 mediated, intergenic |
|  | SNP | Transition | 1,439,673 | 1 | G→A | Ala (623) → Thr | non-synonymous | *adhE* | Alcohol dehydrogenase |  |  |  |
|  | SNP | Transversion | 2,972,858 | 1 | T→G | Met (158) → Arg | non-synonymous | *agn43* | Antigen 43 (Ag43) phase‑variable biofilm formation autotransporter |  |  |  |
|  | Deletion | Large | 2,057,167 | 127 | 2 genes |  |  | *[glnU]* |  | *[glnW]* |  | IS150 mediated |

^a^ Sub-classification of mutation: SNP (transversion, transition), mobile element (insertion, deletion), deletion (small <10 bp, large >11 bp), insertion (small <10, duplication).

^b^ The position on the reference genome at which the mutation occurred. In some cases, particularly with large deletions, the exact location of the beginning of the mutation could not be specified from the breseq output, and instead, an indicative size range is given.

^c^ Frameshifts, gene disruptions and non-synonymous mutations are predicted to change protein function.

^d^ The mutated gene. In the case of genes partially deleted as part of a large multi-gene deletion, these are shown in parentheses [ ]. Intergenic mutations are identified by the gene closest to the mutation, with distance from the beginning of the gene (“+” for downstream and “-“ for upstream) shown in bp.

^e^ For intergenic mutations, the nearest gene downstream of the mutation, Reference Gene 2, is listed, with the distance (in bp) from Reference Gene 2 shown in parentheses.

Supplementary Table 3 List of all mutations in FL lineages

| Lineage | Mutation  class | Mutation  sub-class^a^ | Reference genome position 1^b^ | Size (bp) | Base change | Amino  acid change | Functional effect^c^ | Reference Gene 1^d^ | Gene 1 description | Reference  Gene 2^e^ | Gene 2 description | General comment |
| --- | --- | --- | --- | --- | --- | --- | --- | --- | --- | --- | --- | --- |
| 1 | Deletion | Large | 4,533,440 | 2,214 | 2 genes |  |  | *[yjiY]* | Putative protein | *[hpaC]* | 4-hydroxyphenylacetate 3-monooxygenase reductase component | Mediated by two IS150 insertions |
|  | Mobile element | Insertion | 959,668 | 1,446 |  |  | gene disruption | *btuC* | Vitamin B_12_-transporter permease |  |  | IS150 mediated |
|  | Mobile element | Insertion | 1,288,903 | 768 |  |  | gene disruption | *ydbD* | Hypothetical protein |  |  | IS1 mediated |
|  | Mobile element | Insertion | 1,341,071 | 768 |  |  | gene disruption | *ynaI* | Inner membrane protein |  |  | IS1 mediated |
|  | Mobile element | Insertion | 4,381,583 | 1,472 |  |  | gene disruption | *cycA* | D-alanine, D-serine, glycine permease |  |  | IS150 mediated |
|  | Mobile element | Deletion | 1,764,886 | 1,446 |  |  | gene restoration | *pflB* | Pyruvate formate lyase 1 |  |  | IS150 mediated |
|  | SNP | Transition | 2,229,796 | 1 | A→G |  | non-coding | *mqo*  (-116) | Malate:quinone oxidoreductase | *yojI*  (+102) | Multidrug transporter membrane component | Intergenic |
|  | SNP | Transition | 20,661 | 1 | T→C | Ile (372) → Thr | non-synonymous | *ECB_00021* | Putative usher protein |  |  |  |
|  | Deletion | Large | 4,533,440 | 2,214 | 2 genes |  | partial gene deletion | *[yjiY]* | Putative protein | *[hpaC]* | 4-hydroxyphenylacetate 3-monooxygenase reductase component |  |
| 2 | Deletion | Large | 1,110,292 | 3,055 | 5 genes |  |  | *[ydfX]* |  | *ECB_01533* |  | IS150 mediated |
|  | Mobile element | Insertion | 388,543 | 1,473 |  |  | gene disruption | *brnQ* | Branched chain amino acid transporter |  |  | IS150 mediated |
|  | Mobile element | Insertion | 462,604 | 1,446 |  |  | gene disruption | *priC* | Primosomal replicon protein N for restarting stalled replication forks |  |  | IS150 mediated |
|  | Mobile element | Insertion | 1,580,827 | 1,446 |  |  | gene disruption | *flgK* | Flagellar hook associated protein |  |  | IS150 mediated |
|  | Mobile element | Insertion | 4,381,583 | 1,472 |  |  | gene disruption | *cycA* | D-alanine, D-serine, glycine permease |  |  | IS150 mediated |
|  | Mobile element | Deletion | 429,505 | 1,350 |  |  | gene restoration | *insL-2* | Transposase of IS186 |  |  | IS186 mediated |
|  | Mobile element | Deletion | 1,764,888 | 1,446 |  |  | gene restoration | *pflB* | Pyruvate formate lyase I |  |  | IS150 mediated |
|  | Mobile element | Insertion | 654,734 | 1,472 |  |  | non-coding | *ogrK* (+21) | DNA binding transcriptional regulator | *yegQ*  (+251) | Putative peptidase | IS150 mediated, intergenic |
|  | Mobile element | Insertion | 1,272,468 | 1,446 |  |  | non-coding | *trg*  (-326) | Methyl‑accepting chemotaxis protein III, ribose and galactose sensor receptor | *mokB*  (-13) | Regulatory peptide | IS150 mediated, intergenic |
|  | Mobile element | Insertion | 1,464,678 | 1,446 |  |  | non-coding | *ldrC*  (-343) | *ldrC* is one of a set of four Long Direct Repeats (LDRs), one of which has been shown to code for the toxin portion of a toxin-antitoxin pair. | *ldrB*  (-85) | *ldrB* is one of a set of four Long Direct Repeats (LDRs), one of which has been shown to code for the toxin portion of a toxin-antitoxin pair | IS150 mediated, intergenic |
|  | Mobile element | Insertion | 3,386,643 | 1,446 |  |  | non-coding | *yhfA*  (-114) | Hypothetical protein | *crp*  (-186) | cAMP regulatory protein | IS150 mediated, intergenic |
|  | Mobile element | Insertion | 4,532,958 | 1,473 |  |  | non-coding | *yjiX*  (-31) | Hypothetical protein | *yjiY*  (+19) | Putative inner membrane protein | IS150 mediated, intergenic |
|  | SNP | Transversion | 1,290,872 | 1 | A→G | Lys (285) → Glu | non-synonymous | *insF-2* | IS3 element protein InsF |  |  |  |
|  | Deletion | Large | 1,110,292 | 3,055 | 5 genes |  | partial gene deletion | *[ydfX]* | pseudogene Qin prophage |  |  |  |
| 3 | Mobile element | Insertion | 960,637 | 1,446 |  |  | gene disruption | *btuE* | Putative glutathione peroxidase |  |  | IS150 mediated |
|  | Mobile element | Insertion | 3,000,519 | 1,413 |  |  | gene disruption | *kpsM* | Polysialic acid transport protein KpsM |  |  |  |
|  | Mobile element | Insertion | 4,299,101 | 1,446 |  |  | gene disruption | *lysU* | Lysl-tRNA synthetase |  |  | IS150 mediated |
|  | Mobile element | Insertion | 4,381,583 | 1,472 |  |  | gene disruption | *cycA* | D-alanine, D-serine, glycine permease |  |  | IS150 mediated |
|  | Mobile element | Insertion | 4,534,750 | 1,446 |  |  | gene disruption | *yjiY* | Putative inner membrane protein |  |  | IS150 mediated |
|  | Mobile element | Deletion | 1,764,886 | 1,446 |  |  | gene restoration | *pflB* | Pyruvate formate lyase I |  |  | IS150 mediated |
|  | Insertion | Small | 388,020 | 4 | +ATCA |  | indel frameshift | *brnQ* | Branched chain amino acid transporter |  |  |  |
|  | Insertion | Small | 422,593 | 1 |  |  | non-coding | *cyoA*  (-280) | Cytochrome o ubiquinol oxidase subunit II | *ampG*  (+182) | Muropeptide transporter | Intergenic, slippage likely |
|  | Deletion | Small | 4,240,695 - 4,240,865 | 3,348 |  |  | non-coding | *gltP* | Glutamate and aspartate proton symporter | *yjcO* | Hypothetical protein | Intergenic |
|  | Deletion | Small | 1,328,493 | 37 |  |  | partial gene deletion | *[zntB]* | Trans-membrane zinc transporter |  |  |  |
|  | SNP | Transversion | 1,329,917 | 1 | A→T | Ser (54) → Ser | synonymous | *ydaM* | Putative diguanylate cyclase |  |  |  |
|  | SNP | Transition | 3,829,404 | 1 | G→A | Lys (125) → Lys | synonymous | *yieG* | Putative inner membrane protein |  |  |  |
| 4 | Deletion | Large | 963,078 | 638 | 2 genes |  |  | *[ydiV]* | Hypothetical proteins | *[ydiU]* |  | IS150 mediated |
|  | Mobile element | Insertion | 388,275 | 1,446 |  |  | gene disruption | *brnQ* | Branched chain amino acid transporter |  |  | IS150 mediated |
|  | Mobile element | Insertion | 1,181,538 | 1,446 |  |  | gene disruption | *yddA* | Multidrug ABC transporter membrane ATP binding protein |  |  | IS150 mediated |
|  | Mobile element | Insertion | 2,138,008 | 1,446 |  |  | gene disruption | *yehU* | Putative sensory kinase in two-component system with YehT |  |  | IS150 mediated |
|  | Mobile element | Insertion | 4,381,583 | 1,472 |  |  | gene disruption | *cycA* | D-alanine, D-serine, glycine permease |  |  | IS150 mediated |
|  | Mobile element | Deletion | 1,764,888 | 1,446 |  |  | gene restoration | *pflB* | Pyruvate formate lyase I |  |  | IS150 mediated |
|  | Mobile element | Insertion | 16,972 | 1,446 |  |  | non-coding | *hokC*  (-71) | Small toxic membrane polypeptide | *nhaA*  (-514) | pH dependent sodium or proton antiporter | IS150 mediated, intergenic |
|  | Mobile element | Insertion | 1,551,960 | 1,446 |  |  | non-coding | *ycfQ* (+33) | putative DNA‑binding transcriptional regulator | *ycfJ*  (+29) | Hypothetical protein | IS150 mediated, intergenic |
|  | Mobile element | Insertion | 1,762,790 | 1,446 |  |  | non-coding | *ycaO* (+33) | Hypothetical protein | *focA*  (-370) | Formate transporter | Intergenic |
|  | SNP | Transversion | 3,153,950 | 1 | A→T |  | non-coding | *yhaO*  (-148) | Putative transporter | *tdcG*  (+191) | L-serine dehydratase 3 | Intergenic |
|  | SNP | Transition | 3,041,966 | 1 | A→G | Tyr (439) → His | non-synonymous | *pitB* | Phosphate transporter |  |  |  |
|  | Deletion | Large | 963,078 | 638 | 2 genes |  | partial gene deletion | *[ydiV]* | Involved in motility and quorom sensing | *[ydiU]* | Control of motility, regulate expression of flagella and motility in response to nutrient availability |  |
| 5 | Mobile element | Insertion | 959,872 | 1,446 |  |  | gene disruption | *btuC* | Vitamin B_12_-transporter permease |  |  | IS150 mediated |
|  | Mobile element | Insertion | 4,381,583 | 1,472 |  |  | gene disruption | *cycA* | D-alanine, D-serine, glycine permease |  |  | IS150 mediated |
|  | Mobile element | Insertion | 4,533,508 | 1,473 |  |  | gene disruption | *yjiY* | Putative inner membrane protein |  |  | IS150 mediated |
|  | Mobile element | Deletion | 1,764,888 | 1,446 |  |  | gene restoration | *pflB* | Pyruvate formate lyase I |  |  | IS150 mediated |
|  | Insertion |  | 1,328,521 | 1 | +A |  | indel frameshift | *zntB* | Trans-membrane zinc transporter |  |  |  |
|  | SNP | Transversion | 1,328,511 | 1 | C→A | Ala (57) → Glu | non-synonymous | *trpC* | Indole-3- glycerol phosphate synthase |  |  |  |
|  | SNP | Transition | 4,343,175 | 1 | C→T | Thr (579) → Ile | non-synonymous | *mutL* | DNA mismatch repair |  |  |  |
| 6 | Deletion | Large | 4,532,961 | 19,650 | 16 genes |  |  | *yjiY* | Induced in stationary phase when cells are growing on amino acids or peptides | *[mdoB]* | L-galactonate oxidoreductase that is required for growth on L-galactonate as the sole carbon source under high-throughput growth conditions with limited aeration | IS150 mediated |
|  | Mobile element | Insertion | 360,203 | 1,446 |  |  | gene disruption | *yaiT* | Hypothetical protein |  |  | IS150 mediated |
|  | Mobile element | Insertion | 388,275 | 1,446 |  |  | gene disruption | *brnQ* | Branched chain amino acid transporter |  |  | IS150 mediated |
|  | Mobile element | Insertion | 471,785 | 1,446 |  |  | gene disruption | *aes* | Acetyl esterase |  |  | IS150 mediated |
|  | Mobile element | Insertion | 785,037 | 1,446 |  |  | gene disruption | *cheB* | Chemotaxis-specific methylesterase |  |  | IS150 mediated |
|  | Mobile element | Insertion | 960,637 | 1,446 |  |  | gene disruption | *btuE* | Putative glutathione peroxidase |  |  | IS150 mediated |
|  | Mobile element | Insertion | 974,185 | 1,446 |  |  | gene disruption | *ydiQ* | Putative electron transfer flavoprotein YdiQ |  |  | IS150 mediated |
|  | Mobile element | Insertion | 1,598,705 | 1,446 |  |  | gene disruption | *dinI* | DNA damage-inducible protein |  |  | IS150 mediated |
|  | Mobile element | Insertion | 1,675,380 | 1,446 |  |  | gene disruption | *yccC* | Cryptic autophosphorylating protein tyrosine kinase Etk |  |  | IS150 mediated |
|  | Mobile element | Insertion | 3,972,154 | 1,443 |  |  | gene disruption | *fadA* | 3-ketoacyl-CoA thiolase |  |  | IS150 mediated |
|  | Mobile element | Insertion | 4,381,583 | 1,472 |  |  | gene disruption | *cycA* | D-alanine, D-serine, glycine permease |  |  | IS150 mediated |
|  | Mobile element | Deletion | 1,764,888 | 1,446 |  |  | gene restoration | *pflB* | Pyruvate formate lyase I |  |  | IS150 mediated |
|  | Insertion | Small | 1,328,521 | 1 | +A |  | indel frameshift | *zntB* | Trans-membrane zinc transporter |  |  |  |
|  | Mobile element | Insertion | 1,272,468 | 1,446 |  |  | non-coding | *trg*  (-326) | Methyl accepting chemotaxis protein III | *mokB*  (-13) | Regulatory peptide | IS150 mediated, intergenic |
|  | Deletion | Small | 3,543,375 | 1 |  |  | non-coding | *yhiO*  (-160) | Universal stress protein UspB | *uspA*  (-231) | Universal stress global regulator | Intergenic |
| 7 | Deletion | Large | 380,365 | 7,461 | 6 gene |  |  | *[araJ]* | Sugar efflux system | *[brnQ]* | Putative branched chain amino acid transporter |  |
|  | Deletion | Large | 546,986 – 547,703 | 4,157 – 4,874 | 7 genes |  |  | *[insB-6]* | IS150 protein | *[ompT]* |  | IS1 mediated |
|  | Mobile element | Insertion | 963,716 | 1,473 |  |  | gene disruption | *ydiU* | Hypothetical protein |  |  | IS150 mediated |
|  | Mobile element | Insertion | 974,185 | 1,446 |  |  | gene disruption | *ydiQ* | Putative electron transfer flavoprotein YdiQ |  |  | IS150 mediated |
|  | Mobile element | Insertion | 2,628,603 | 1,446 |  |  | gene disruption | *yfiH* | Hypothetical protein |  |  | IS150 mediated |
|  | Mobile element | Insertion | 2,654,657 | 777 |  |  | gene disruption | *ECB_02512* | Hypothetical protein |  |  | IS1 |
|  | Mobile element | Insertion | 4,239,784 | 1,473 |  |  | gene disruption | *gltP* | Glutamate/aspartate proton symporter |  |  | IS150 mediated |
|  | Mobile element | Insertion | 4,381,583 | 1,462 |  |  | gene disruption | *cycA* | D-alanine, D-serine, glycine permease |  |  | IS150 mediated |
|  | Mobile element | Deletion | 1,764,886 | 1,446 |  |  | gene restoration | *pflB* | Pyruvate formate lyase I |  |  | IS150 mediated |
|  | Mobile element | Insertion | 16,989 | 1,489 |  |  | non-coding | *hokC*  (-88) | Small toxic membrane polypeptide | *nhaA*  (-496) | pH dependent sodium or proton antiporter | IS150 mediated, intergenic |
|  | Mobile element | Insertion | 582,237 | 1,446 |  |  | non-coding | *insA-7* (+193) | IS1 protein | *hokE* | toxic polypeptide, small | IS150 mediated, intergenic |
|  | Mobile element | Insertion | 1,272,468 | 1,446 |  |  | non-coding | *trg*  (-326) | Methyl accepting chemotaxis protein III | *mokB*  (-13) | Regulatory peptide | IS150 mediated, intergenic |
|  | Mobile element | Insertion | 1,464,061 | 1,446 |  |  | non-coding | *chaA* (+238) | calcium/sodium:proton antiporter | *ldrC*  (-167) | *ldrC* is one of a set of four Long Direct Repeats (LDRs), one of which has been shown to code for the toxin portion of a toxin-antitoxin pair. | IS150 mediated, intergenic |
|  | Mobile element | Insertion | 4,382,959 | 1,472 |  |  | non-coding | *yjiX*  (-32) | Hypothetical protein | *yjiY*  (+17) | Putative inner membrane protein | IS150 mediated, intergenic |
|  | SNP | Transition | 2,069,532 | 1 | C→T | Ser (176) → Phe | non-synonymous | *gltK* | Glutamate and aspartate transporter subunit |  |  |  |

^a^ Sub-classification of mutation: SNP (transversion, transition), mobile element (insertion, deletion), deletion (small <10 bp, large >11 bp), insertion (small <10, duplication).

^b^ The position on the reference genome at which the mutation occurred. In some cases, particularly with large deletions, the exact location of the beginning of the mutation could not be specified from the breseq output, and instead, an indicative size range is given.

^c^ Frameshifts, gene disruptions and non-synonymous mutations are predicted to change protein function.

^d^ The mutated gene. In the case of genes partially deleted as part of a large multi-gene deletion, these are shown in parentheses [ ]. Intergenic mutations are identified by the gene closest to the mutation, with distance from the beginning of the gene (“+” for downstream and “-“ for upstream) shown in bp.

^e^ For intergenic mutations, the nearest gene downstream of the mutation, Reference Gene 2, is listed, with the distance (in bp) from Reference Gene 2 shown in parentheses.
